# Effects of physical activity and sedentary time on depression, anxiety and well-being: a bidirectional Mendelian randomisation study

**DOI:** 10.1186/s12916-023-03211-z

**Published:** 2023-12-18

**Authors:** Francesco Casanova, Jessica O’Loughlin, Vasilis Karageorgiou, Robin N. Beaumont, Jack Bowden, Andrew R. Wood, Jessica Tyrrell

**Affiliations:** Genetics of Complex Traits, Department of Biomedical & Clinical Science, University of Exeter Medical School, Exeter, UK

**Keywords:** Mental health, Well-being, Physical activity, Mendelian randomisation

## Abstract

**Background:**

Mental health conditions represent one of the major groups of non-transmissible diseases. Physical activity (PA) and sedentary time (ST) have been shown to affect mental health outcomes in opposite directions. In this study, we use accelerometery-derived measures of PA and ST from the UK Biobank (UKB) and depression, anxiety and well-being data from the UKB mental health questionnaire as well as published summary statistics to explore the causal associations between these phenotypes.

**Methods:**

We used MRlap to test if objectively measured PA and ST associate with mental health outcomes using UKB data and summary statistics from published genome-wide association studies. We also tested for bidirectional associations. We performed sex stratified as well as sensitivity analyses.

**Results:**

Genetically instrumented higher PA was associated with lower odds of depression (OR = 0.92; 95% CI: 0.88, 0.97) and depression severity (beta =  − 0.11; 95% CI: − 0.18, − 0.04), Genetically instrumented higher ST was associated higher odds of anxiety (OR = 2.59; 95% CI: 1.10, 4.60). PA was associated with higher well-being (beta = 0.11, 95% CI: 0.04; 0.18) and ST with lower well-being (beta =  − 0.18; 95% CI: − 0.32, − 0.03). Similar findings were observed when stratifying by sex. There was evidence for a bidirectional relationship, with higher genetic liability to depression associated with lower PA (beta =  − 0.25, 95% CI: − 0.42; − 0.08) and higher well-being associated with higher PA (beta = 0.15; 95% CI: 0.05, 0.25).

**Conclusions:**

We have demonstrated the bidirectional effects of both PA and ST on a range of mental health outcomes using objectively measured predictors and MR methods for causal inference. Our findings support a causal role for PA and ST in the development of mental health problems and in affecting well-being.

**Supplementary Information:**

The online version contains supplementary material available at 10.1186/s12916-023-03211-z.

## Background

Mental health conditions are a significant contributor to the burden of non-transmissible diseases worldwide. They pose a significant challenge to patients and healthcare systems, with a considerable impact on the global economy, costing trillions of dollars per year [1]. In 1946, the World Health Organisation (WHO) re-defined “health” as a state of complete physical, mental, and social well-being and not merely the absence of disease or infirmity. The concept of health is, therefore, extended to quality of life with emphasis on a person’s mental health and general well-being. Understanding factors that contribute to poorer mental health and lower well-being is crucial to ensure appropriate public health prevention strategies and messaging.

Higher levels of physical activity (PA) have been found to be associated with improved mental health and well-being [2–7] whilst sedentary time (ST) increases the risk of depression and anxiety [8, 9] and contributes to lower emotional well-being [10, 11]. ST, defined as time spent performing activities of less than 1.5 metabolic equivalent units such as sitting or lying down while awake [12], has received increasing attention in recent years as an independent predictive risk factor for disease [13–15] and it is believed to be conceptually different from low PA [16].

Most evidence for the effects of PA and ST on mental health and well-being comes from small to medium size exercise intervention trials or observational studies that suffer from potential unmeasured biases, even when well-designed. Randomised control trials (RCTs) are the gold standard for exploring causality. However, large-scale RCTs cannot always be performed because they can be costly, impractical, or even unethical [17]. Mendelian randomisation (MR) is a genetic approach that is similar to RCTs in terms of study design. It is extensively described elsewhere [17]; briefly, it utilises the random distribution of alleles at birth [17] to infer causality. MR uses genetic variants as instrumental variables for modifiable risk factors that affect population health. The method can overcome some of the limitations of conventional observational studies including confounding and reverse causation [18]. A recent MR study in the UK Biobank (UKB) utilised genetic variants that were associated with accelerometery-derived PA to infer a causal relationship between PA and depression [19] but provided no evidence of a causal association of depression on PA (i.e. no bidirectionality). Exploring bidirectionality is important to tease apart the relationships between PA, ST and mental health and well-being [20].

To the best of our knowledge, no MR studies have investigated the association between PA and other mental health phenotypes such as anxiety and well-being. Similarly, no previous MR study has investigated the association between ST and mental health and well-being outcomes. Until recently there was no suitable ST dataset for MR. Using a machine learning algorithm Doherty et al. [21] analysed accelerometery data in approximately 100,000 participants from UKB and classified activity into overall PA, sleep and ST. These data allow us to consider objectively measured ST, as well as PA, and mental health outcomes using MR techniques providing the advantage of homogeneity in data collection and outcome definitions, as well as the ability to explore casual associations at the population level.

In this study, we used MR to investigate the effects the accelerometery-derived measures of PA and ST from the UKB as exposures and data from the UKB mental health questionnaire (MHQ) as well as published GWAS of depression, anxiety and well-being as outcomes [22–24]. We accounted for several potential sources of bias, tested for bidirectional associations (i.e. mental health is causally associated with PA and ST), and performed sex-stratified analyses due to the different incidence of mental health problems between males and females [25].

## Methods

### Population

We used 451,025 individuals of European ancestry (defined through principal component analyses [26]) from the UKB study [27]. UKB recruited over 500,000 individuals and collected detailed information from all participants, via questionnaires, interviews and measurements.

### Exposures and outcomes

#### Physical activity (PA) and sedentary time (ST)

In UKB PA was objectively measured using accelerometery data in 95,776 European individuals. We derived overall PA as described by Doherty et al. [21], we used the mean average vector magnitude for each 30-s epoch over the 7 days wear time.

We derived ST from accelerometery data using a machine learning algorithm (https://github.com/activityMonitoring/biobankAccelerometerAnalysis) as described elsewhere [21, 28]. Briefly, for every non-overlapping 30-s time window, the algorithm extracts 126-dimensional feature vectors representing a range of time and frequency domain features. These vectors are then used to classify activities in each 30-s window into sedentary (used here to perform the ST GWAS analysis) and other activities (not used here) using a random forest nonparametric discrimination model. The predictions are then smoothed using a hidden Markov model.

Genetic associations for inverse-normalised PA and ST were tested using a linear mixed model approach with BOLT-LMM [29]. These were adjusted for age, sex, study centre, and genotyping array. Variants with imputation quality (INFO) < 0.3 or minor allele frequency < 1% were excluded.

As no genome-wide (*p* < 5 × E − 08) SNPs were found for ST and only three were below this threshold for PA, the *p*-value threshold for SNPs used in our analysis was relaxed to 1 × E − 05 to maximise the number of SNPs in our instruments. Sensitivity analysis at different *P*-value thresholds was also performed (see below). To obtain independent SNPs to use as genetic instruments in the MR analyses, the full summary statistics from the GWAS analyses were clumped using a distance of 1 Mb and an R^2^ threshold of 0.001 (Additional file 1: Table S1 and S2).

#### Depression, anxiety and well-being

For mental health metrics, we first focused on using published summary statistics from the largest available GWAS as genetic instruments and we then used the mental health questionnaire (MHQ) data in UK Biobank, to enable sex-specific analyses.

Firstly, we used genome-wide significant SNPs from published mental health GWAS [22–24] as instruments for the exposures (Additional file 1: Tables S3–5). For depression, we used summary statistics from the Psychiatric Genomic Consortium (PGC) [22] (*n* = 1,306,354; 414,055 cases), excluding 23andMe (these data are not shared by PGC because of transfer agreement restrictions; Additional file 1: Table S3). We did not have access to summary statistics from the most recent depression GWAS [30].

For anxiety, we also used PGC summary statistics [23] (*n* = 21,761; 7016 cases; Additional file 1: Table S4). For well-being, we used summary statistics from Okbay et al. [24] which measured subjective well-being as life satisfaction, and positive affect in 298,420 individuals (Additional file 1: Table S5).

Secondly, we derived the mental health outcomes from the UKB MHQ. A total of 145,668 individuals completed the MHQ and we derived depression, anxiety and wellbeing using freely available R code (https://data.mendeley.com/datasets/kv677c2th4/3), as described elsewhere [31]. More information on the mental health variables derived is briefly below and in the supplement (Additional file 1: Methods).

#### Depression

Depression was assessed using the Composite International Diagnostic Interview Short Form (CIDI-SF) and the Patient Health Questionnaire-9 (PHQ9) questionnaires. From the CIDI-SF we derived a binary measure of lifetime major depression and a continuous variable for the severity of lifetime depression. Using PHQ9 we derived both a binary measure of current depression and a continuous variable for the severity of current depression [22, 30].

#### Anxiety

Anxiety was assessed using the Generalised Anxiety Disorder 7 (GAD-7) item questionnaire. Based on this assessment, we derived two binary anxiety variables: current GAD and lifetime GAD. Additionally, we created a continuous variable to represent GAD severity[23].

#### Well-being

A well-being score was derived from three variables that made up part of the MHQ [31]. Two questions assessed the subjective, or hedonic, aspect of well-being: “general happiness” (20,458) and “happiness with health” (20,459). The third question, taken from the WHO-Quality of Life, measured the eudaimonic, or psychological aspect of well-being: “belief that my life is meaningful” (20,460).

Each of the three variables was assessed individually and summed to provide an overall ‘well-being score’ for 141,829 participants.

### Analysing associations between exposures and outcomes

#### Observational association analysis in the UK Biobank

Linear (continuous) and logistic (binary) regression models were used to test observational associations between PA, ST and our mental health metrics. Models were adjusted for age and sex, and further adjusted for body mass index (BMI) and socioeconomic status (Townsend deprivation index, TDI). Sex stratified analyses were also performed.

#### Mendelian randomisation analysis

For our MR analyses, we used MRlap (https://github.com/n-mounier/MRlap) [32]. This is a relatively new method, that considers a number of biases that MR analyses can be subject to. MRlap corrects for weak instrument bias and winner’s curse, whilst accounting for sample overlap and its effect as a modifier of these biases. The authors introduced an analytical derivation of the expected value of the standard IVW causal effect estimate, assuming a spike-and-slab genomic architecture for the exposure. The standard IVW estimate is equivalent to a weighted regression of the SNP-outcome association estimates on the SNP-exposure association estimates constraining the intercept to zero. The estimated regression coefficient represents the standard deviation (SD) change in the outcome per SD change in the exposure variable, with the exception of binary outcomes where it represents log(odds ratio). The IVW causal effect expectation relies solely on the true underlying causal effect and parameters that can be estimated from GWAS summary statistics. These parameters include the cross-trait LD-score intercept, which is proportionate to the degree of sample overlap, as well as the heritability and polygenicity of the exposure. Consequently, it becomes feasible to adjust the IVW estimate and propose a corrected effect estimate that remains robust against weak instrument bias and winner's curse, regardless of the degree of sample overlap between the exposure and outcome samples.

MRlap calculates a test statistic to highlight if the corrected effect estimate significantly differs from the IVW observed effect. If there is no difference, then the IVW-MR estimate can be safely used. However, when there is a significant difference, corrected effects should be preferred as they should be less biased, independently of the sample overlap. This method relies on the same instrumental variable assumptions (relevance, exclusion restriction and independence assumptions) that IVW and therefore could be biased in the presence of correlated pleiotropy. Moreover, to be able to correctly estimate the genomic architecture parameters, the spike-and-slab assumption must hold, and the approach does not work well for traits that are not heritable or not polygenic enough. Finally, when working with case–control data, the sample overlap between the exposure and the outcome data is assumed to be the same for both cases and controls. Detailed information about the method and its approach to adjusting for biases can be found in Mounier et al. [32].

This method was appropriate for our analyses, as we had:

A) Sample overlap between our PA/ST metrics and mental health, even when using the published summary statistics. For example, within UK Biobank there is a 46% overlap between the accelerometery and MHQ subsets of UKB;
B) Weak instruments when using PA/ST as exposures;
C) Winner’s curse as our instruments for PA/ST were derived in the same study as our outcomes;

All MR analyses rely on several assumptions [32]:

- The exposure SNPs are robustly associated with the relevant measured exposure. This is quantified by the F-statistic, which can be approximated by the ratio of the SNP-exposure association estimate, $\widehat{\beta }$ and its standard error, $SE(\widehat{\beta })$, squared (Eq. 1) [33].
  1 $$\mathrm{F }= {\left(\frac{\widehat{\beta }}{{SE(\widehat{\beta })}}\right)}^{2}$$
- The exposure SNPs are not associated with confounding factors that bias conventional epidemiological associations.
- The exposure SNPs are only associated with the outcome through the risk factor.

#### Bidirectional analyses

To test for a bidirectional relationship between PA or ST with depression, anxiety or well-being we used MRlap, as described above, using the latest available PGC summary statistics for depression [22], anxiety [23], subjective well-being [24] and the UKB MHQ well-being score as exposures and PA and ST as outcomes.

#### Sex stratified analysis

To test the hypothesis that the effects of PA and ST on mental health differ between males and females we ran sex-specific GWAS for our mental health outcomes and formally compared the association estimates using Fisher’s *z* score (Eq. 2). Using Eq. 2, we also tested if the effects of depression, anxiety and well-being on PA and ST differed between males and females.

2 $$z= \frac{\widehat{{\beta }_{male}}- \widehat{{\beta }_{female}}}{\sqrt{{SE(\widehat{{\beta }_{male}})}^{2}+{SE(\widehat{{\beta }_{female}})}^{2}}}$$

#### Sensitivity analysis

1. Analysis excluding known depression and anxiety loci

We excluded all PA and ST loci also known to be depression and anxiety loci, defined as reaching genome-wide significance in the primary GWAS. Depression and anxiety loci were taken from the most recent GWAS studies [22, 23, 30, 34–36]. PA or ST SNPs were removed from analysis if the SNP was in linkage disequilibrium (defined as *R*^2^ > 0.1) with a depression, anxiety or well-being SNP in the same locus (defined as distance < 500 kb). Linkage disequilibrium was determined using a freely available online tool (https://ldlink.nci.nih.gov/?tab=ldpair) using the European reference population.

2. Analysis using other MR methods, including pleiotropy robust methods

Four 2-sample MR methods were performed using a custom pipeline: inverse-variance weighting (IVW); MR-Egger; weighted median (WM); penalised weighted median (PWM). More details of these methods can be found in Additional file 1.

3. Analysis using different *p*-value thresholds for MR instrument selection

When an association was identified, we tested whether the selection of PA and ST instruments, based on a *p*-value threshold of 1 × E − 05, influenced our results. To assess this, we repeated our analyses using stricter *p*-value thresholds (5 × E − 06 and 1 × E − 06).

4. Analysis of the PA and ST instrument using the Wray et al., depression summary statistics to remove bias due to sample overlap

We extracted the genetic variants used as the PA and ST instrument from the older Wray et al., PGC GWAS of major depression that excluded UK Biobank and performed standard 2-sample MR including IVW, MR-Egger, WM and PWM. This analysis limits any bias due to sample overlap.

5. Analysis of individual MHQ questions used to create the well-being score

To further understand how different dimensions of well-being affect our results we performed an MRlap analysis of the individual components of the well-being score (see “Well-being” section above for details of the questions analysed).

## Results

The demographics of individuals with measured mental health outcomes are summarised in Table 1. Briefly, depression and anxiety were more prevalent in females, with females also reporting more severe symptoms. No sex differences were observed for well-being.

**Table 1:** Basic demographics of the UK Biobank study participants, data are reported as means (standard deviation) or median [interquartile range]

Trait	All	Females	Males
N	145,982	82,437	63,545
Age (years)	56.56 (7.70)	56.08 (7.63)	57.19 (7.75)
Body mass index (kg/m^2^)	26.78 (4.55)	26.35 (4.88)	27.33 (4.01)
Townsend deprivation index	− 1.79 (2.78)	− 1.75 (2.77)	− 1.83 (2.80)
Sedentary time (hours/day)	9.18 (2.35)	8.89 (2.24)	9.55 (2.43)
Major depression — *N* cases (%)	34,858 (23.88)	24,022 (29.14)	10,836 (17.05)
Current depression — *N* cases (%)	2659 (1.82)	1691 (2.05)	968 (1.52)
Generalised anxiety disorder — *N* cases (%)	7244 (4.96)	4706 (5.71)	2536 (3.99)
Current generalised anxiety disorder — *N* cases (%)	1854 (1.27)	1205 (1.46)	649 (1.02)
Severity of major depression	3 [6]	4 [6]	0 [5]
Severity of current depression	2 [4]	2 [4]	1 [3]
Severity of generalised anxiety disorder	0 [3]	1 [4]	0 [2]
Well-being score	13 [3]	13 [3]	13 [3]

### Observational associations in UK Biobank MHQ

Observationally, higher PA was associated with lower odds of major and current depression as well as lower odds of current and lifetime GAD (Additional file 1: Table S6). For example, a 1-SD higher PA was associated with lower odds of major depression (Odds Ratio (OR): 0.85, 95% confidence intervals (CI): 0.83;0.86). Further, higher PA was associated with higher well-being scores and lower depression and anxiety severity. Further adjustment for BMI and TDI did not change the results (Additional file 1: Table S6).

Higher ST was observationally associated with higher odds of major and current depression, as well as more severe depression and lower well-being (Additional file 1: Table S6). No association was observed between ST and anxiety. Further adjustment for BMI and TDI attenuated the association for current and lifetime.

### Our MR analyses used valid instruments

The final SNPs used as instruments can be found in Additional file 1: Tables S1–5. Mean F-statistics for these SNPs ranged between 17.7 and 41.2, providing evidence that our exposure SNPs were robustly associated with the relevant measured exposure. We summarised known associations of our exposure variants (Additional file 1: Tables S1–5) and tested for potential associations with potential confounders using 2-sample MR. The PA instrument was nominally associated with BMI and lower odds of ever smoking, although not when using more pleiotropy robust methods like MR Egger. The ST instrument was nominally associated with educational attainment. No association was noted with other confounders (alcohol consumption, BMI, diet, educational attainment and smoking).

### Genetically instrumented higher PA was associated with lower odds and severity of depression whilst ST was not associated with depression

**Fig. 1:**
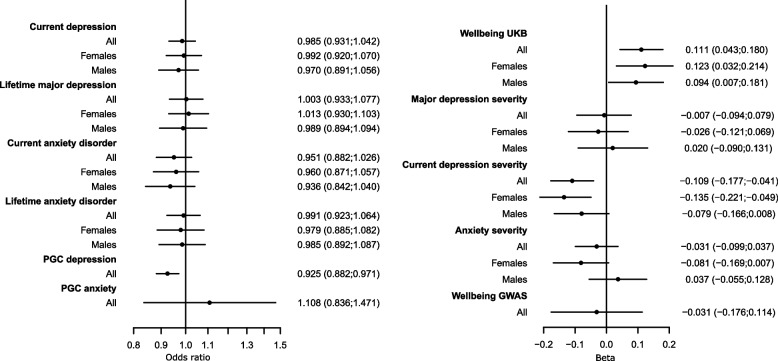
Forest plot of the results of the Mendelian randomisation analysis (MRlap) using genetically instrumented physical activity as exposure and binary (left) and continuous (right) outcome. Data represent standard deviation change in outcome per standard deviation change in exposure

When using the larger PGC dataset, a genetically instrumented 1-SD higher PA was associated with 0.92 lower odds of major depression (95% CI: 0.88; 0.97) (Table 2 and Fig. 1). Furthermore, a genetically instrumented 1-SD higher PA was associated with lower current depression severity (beta =  − 0.11; 95% CI: − 0.18; − 0.04). Using UKB-derived mental health measures only, there was no evidence for an association between PA and current and major depression, or lifetime depression severity (Table 2 and Fig. 1).

**Table 2:** Results of the 2 sample Mendelian randomisation analysis using MRLap for mental health outcome for all participants and stratified by sex. Results represent odds ratio or betas per standard deviation change in genetically instrumented physical activity

**Exposure**	**Outcome**	**Strata**	**OR (95% CI)**	***P***
Physical activity	PGC-Depression	All	0.93 (0.88; 0.97)	1.58E − 03
Physical activity	PGC-Anxiety	All	1.11 (0.84; 1.47)	4.75E − 01
**Exposure**	**Outcome**	**Strata**	**OR (95% CI)**	***P***
Physical activity	Current depression	All	0.99 (0.93; 1.04)	5.91E − 01
		Females	0.99 (0.92; 1.07)	8.37E − 01
		Males	0.97 (0.89; 1.06)	4.81E − 01
Physical activity	Lifetime major depression	All	1.00 (0.93; 1.08)	9.44E − 01
		Females	1.01 (0.93; 1.10)	7.69E − 01
		Males	0.99 (0.89; 1.09)	8.24E − 01
Physical activity	Current anxiety disorder	All	0.95 (0.88; 1.03)	1.93E − 01
		Females	0.96 (0.87; 1.06)	4.07E − 01
		Males	0.94 (0.84; 1.04)	2.18E − 01
Physical activity	Lifetime anxiety disorder	All	0.99 (0.92; 1.06)	7.99E − 01
		Females	0.98 (0.89; 1.08)	6.78E − 01
		Males	0.99 (0.89; 1.09)	7.64E − 01
**Exposure**	**Outcome**	**Strata**	**Beta (SE)**	***P***
Physical activity	Well-being	All	0.11 (0.04; 0.18)	1.41E − 03
		Females	0.12 (0.03; 0.21)	8.32E − 03
		Males	0.09 (0.01; 0.18)	3.40E − 02
Physical activity	Severity of major depression	All	− 0.01 (− 0.09; 0.08)	8.68E − 01
		Females	− 0.03 (− 0.12; 0.07)	5.92E − 01
		Males	0.02 (− 0.09; 0.13)	7.18E − 01
Physical activity	Severity of current depression	All	− 0.11 (− 0.18; − 0.04)	1.60E − 03
		Females	− 0.14 (− 0.22; − 0.05)	2.18E − 03
		Males	− 0.08 (− 0.17; 0.01)	7.67E − 02
Physical activity	Severity of anxiety	All	− 0.03 (− 0.10; 0.04)	3.65E − 01
		Females	− 0.08 (− 0.17; 0.01)	7.09E − 02
		Males	0.04 (− 0.06; 0.13)	4.29E − 01
Physical activity	Subjective well-being (GWAS)	All	− 0.03 (− 0.18; 0.11)	6.74E − 01

**Fig. 2:**
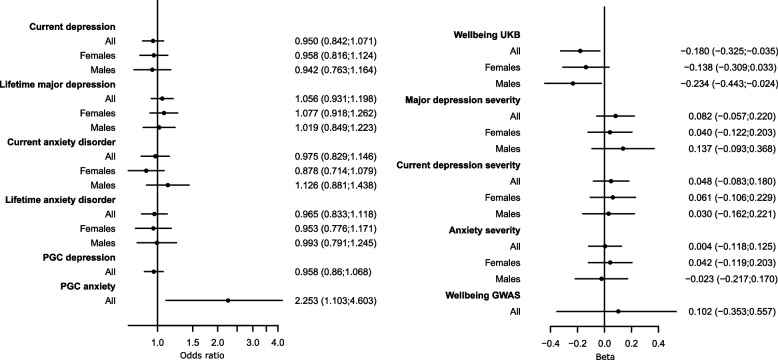
Forest plot of the results of the Mendelian randomisation analysis (MRlap) using genetically instrumented sedentary time as exposure and binary (left) and continuous (right) outcome. Data represent standard deviation change in outcome per standard deviation change in exposure

Genetically instrumented higher ST was not associated with either the PGC summary statistics for major depression nor the UKB binary or continuous measures of major and current depression (Table 3 and Fig. 2).

**Table 3:** Results of the 2 sample Mendelian randomisation analysis using MRLap for mental health outcome for all participants and stratified by sex. Results represent odds ratio or betas per standard deviation change in genetically instrumented sedentary time

**Exposure**	**Outcome**	**Strata**	**OR (95% CI)**	***P***
Sedentary time	PGC-Depression	All	0.96 (0.86; 1.07)	4.38E − 01
Sedentary time	PGC-Anxiety	All	2.25 (1.10; 4.60)	2.59E − 02
**Exposure**	**Outcome**	**Strata**	**Odds ratio (95% CI)**	***P***
Sedentary time	Current depression	All	0.95 (0.84; 1.07)	4.01E − 01
		Females	0.96 (0.82; 1.12)	5.97E − 01
		Males	0.94 (0.76; 1.16)	5.80E − 01
Sedentary time	Lifetime major depression	All	1.06 (0.93; 1.20)	3.97E − 01
		Females	1.08 (0.92; 1.26)	3.62E − 01
		Males	1.02 (0.85; 1.22)	8.41E − 01
Sedentary time	Current anxiety disorder	All	0.98 (0.83; 1.15)	7.55E − 01
		Females	0.88 (0.71; 1.08)	2.15E − 01
		Males	1.13 (0.88; 1.44)	3.43E − 01
Sedentary time	Lifetime anxiety disorder	All	0.97 (0.83; 1.12)	6.34E − 01
		Females	0.95 (0.78; 1.17)	6.47E − 01
		Males	0.99 (0.79; 1.25)	9.50E − 01
**Exposure**	**Outcome**	**Strata**	**Beta (SE)**	***P***
Sedentary time	Well-being	All	− 0.18 (− 0.33; − 0.04)	1.46E − 02
		Females	− 0.14 (− 0.31; 0.03)	1.14E − 01
		Males	− 0.23 (− 0.44; − 0.02)	2.91E − 02
Sedentary time	Severity of major depression	All	0.08 (− 0.06; 0.22)	2.47E − 01
		Females	0.04 (− 0.12; 0.20)	6.26E − 01
		Males	0.14 (− 0.09; 0.37)	2.43E − 01
Sedentary time	Severity of current depression	All	0.05 (− 0.08; 0.18)	4.69E − 01
		Females	0.06 (− 0.11; 0.23)	4.73E − 01
		Males	0.03 (− 0.16; 0.22)	7.60E − 01
Sedentary time	Severity of anxiety	All	0.004 (− 0.12; 0.13)	9.52E − 01
		Females	0.04 (− 0.12; 0.20)	6.12E − 01
		Males	− 0.02 (− 0.22; 0.17)	8.12E − 01
Sedentary time	Subjective well-being (GWAS)	All	0.10 (− 0.35; 0.56)	6.59E − 01

### Genetically instrumented higher PA was not associated with anxiety, whilst ST associated with lower odds of anxiety

MR provided no evidence of an association between higher genetically instrumented PA and current or lifetime GAD (OR: 1.11; 95% CI: 0.84; 1.47) (Table 2 and Fig. 1) using both PGC summary statistics and UKB MHQ-derived measures. Further, there was no association with GAD severity (Table 2 and Fig. 1).

In contrast, genetically instrumented higher ST was associated with higher odds of lifetime anxiety (OR = 2.25; 95% CI: 1.10; 4.60), when using the larger PGC summary statistics (Table 3 and Fig. 2). Using UKB-derived mental health measures only, there was no association with current and lifetime GAD, or GAD severity (Table 3 and Fig. 2).

### Genetically instrumented higher PA and ST was associated with well-being in opposite directions

A 1-SD higher genetically instrumented PA was associated with higher a well-being score (beta = 0.11, 95% CI: 0.04; 0.18, Table 2 and Fig. 2), using the UKB MHQ derived measures. In contrast, there was no association between PA and subjective well-being, which captures life satisfaction and positive affect, from the published GWAS (OR: − 0.031; 95% CI − 0.176; 0.114, Table 2 and Fig. 1).

Similarly, a 1-SD higher ST was associated with a lower well-being score (beta =  − 0.18, 95% CI: − 0.33; − 0.04; Table 3 and Fig. 2) using the MHQ definition from UKB, but there was no association when analysing subjective well-being from the published GWAS (Table 3 and Fig. 2).

### Bidirectional results

A higher genetic liability to depression was associated with lower PA (beta =  − 0.25, 95% CI: − 0.42; − 0.08; Table 4) but not ST (beta = 0.04, 95%C I: − 0.11; 0.18; Table 4). A higher genetic liability to anxiety was not associated with either PA or ST (Table 4).

**Table 4:** Results of the 2 sample Mendelian randomisation analysis using depression, anxiety and well-being as predictors for all participants and stratified by sex. Results represent betas per standard deviation change in genetically instrumented risk of of the exposures

**Exposure**	**Outcome**	**Strata**	**Beta (95% CI)**	**P**
Depression	Overall physical activity	All	− 0.25 (− 0.42; − 0.08)	4.07E − 03
		Females	− 0.45 (− 0.67; − 0.23)	6.05E − 05
		Males	− 0.08 (− 0.27; 0.11)	4.21E − 01
Depression	Sedentary time	All	0.04 (− 0.11; 0.18)	6.33E − 01
		Females	0.17 (− 0.03; 0.36)	9.32E − 02
		Males	− 0.08 (− 0.28; 0.12)	4.13E − 01
**Exposure**	**Outcome**	**Strata**	**Beta (95% CI)**	**P**
Anxiety	Overall physical activity	All	0.00 (− 0.08; 0.08)	9.48E − 01
		Females	0.01 (− 0.10; 0.10)	8.24E − 01
		Males	− 0.01 (-0.11; 0.10)	8.99E − 01
Anxiety	Sedentary time	All	− 0.02 (− 0.13; 0.08)	9.99E − 01
		Females	− 0.04 (− 0.18; 0.09)	5.34E − 01
		Males	0.00 (− 0.13; 0.12)	9.46E − 01
**Exposure**	**Outcome**	**Strata**	**Beta (95% CI)**	**P**
Well-being GWAS	Overall physical activity	All	0.13 (− 0.06; 0.31)	1.68E − 01
		Females	0.07 (− 0.17; 0.31)	5.71E − 01
		Males	0.16 (− 0.04; 0.36)	1.21E − 01
Well-being GWAS	Sedentary time	All	0.07 (− 0.09; 0.23)	3.87E − 01
		Females	0.05 (− 0.18; 0.28)	6.54E − 01
		Males	0.10 (− 0.10; 0.29)	3.27E − 01
Well-being from UKB	Overall physical activity	All	0.15 (0.05; 0.25)	4.65E − 03
		Females	0.18 (0.04; 0.33)	1.33E − 02
		Males	0.12 (− 0.002; 0.24)	5.40E − 02
Well-being from UKB	Sedentary time	All	− 0.02 (− 0.12; 0.09)	7.14E − 01
		Females	− 0.05 (− 0.19; 0.09)	4.75E − 01
		Males	0.00 (− 0.12; 0.12)	9.87E − 01

A genetically instrumented higher well-being using the published summary statistics was not associated with PA (beta = 0.13; 95% CI − 0.06; 0.31, Table 4). However, there was an association when using the UKB MHQ-derived measures with a higher well-being score associating with increased PA (beta = 0.15; 95% CI 0.05; 0.25; Table 4).

There was no association between well-being and ST when using either the published GWAS or the MHQ definition from the UKB (Table 4).

### Sex-stratified analyses

There was no evidence of differences between males and females in our sex-stratified analyses evidenced using Fisher’s *z* score except when using depression as an exposure and PA as an outcome, where the effect was significantly stronger in females than males (Additional file 1: Table S7).

### Sensitivity analyses

- 1. Excluding known loci

We excluded 7 SNPs for PA and 1 for ST (Additional file 1: Tables S1–2). Excluding known depression, anxiety and well-being variants from our PA instrument slightly attenuated our findings for the well-being score and severity of current depression in males (Additional file 1: Table S8). Wider confidence intervals were observed in all other analyses. Excluding depression anxiety and well-being variants from our ST instrument did not substantially change our results (Additional file 1: Table S8). Similarly, our findings did not change when we excluded PA and ST loci from the depression instrument and well-being (Additional file 1: Table S8).

- 2. IVW, Egger and weighted median

Results of the analysis using IVW, MR Egger and weighted median can be found in Additional file 1: Tables S9–10. For PA and ST results of the pleiotropy robust methods are generally in agreement with the results from MRlap, with Egger and weighted median analysis showing directionally consistent results.

- 3. Different *p*-value thresholds

Using *p* = 5E − 06 as our instrument selection threshold for PA, 66 SNPs remained in our instrument. Results for well-being score and severity of current depression were all directionally consistent with 3 out 6 remaining at *P* < 0.05 (Additional file 1: Table S11). Using *p* = 1E − 06 as our instrument selection threshold for PA, 24 SNPs remained in our instrument, with 4 out of 6 remaining directionally consistent but none reaching nominal significance at *P* < 0.05.

Using *p* = 5E − 06 and *p* = 1E − 06 as our instrument selection threshold for ST, 18 and 9 SNPs remained in our instrument, respectively. Results for the well-being score were all directionally consistent but none were nominally significant (*P* < 0.05).

- 4. 2-sample MR with non-overlapping depression GWAS

Using the PA and ST instruments identified in our MRLap analysis we provide further evidence for the role of PA in depression using the summary statistics from the Wray et al. GWAS of major depression excluding the UK Biobank data. Here, a genetically instrumented SD with higher PA was associated with 0.82 lower odds of depression (95% CI: 0.74, 0.92). Results were consistent with more pleiotropy robust methods (Additional file 1: Table S12) and Egger MR did not provide evidence of horizontal pleiotropy (*P*_*intercept*_ = 0.63). In contrast and consistent with our MR lap results genetically instrumented, ST did not predict depression using the Wray et al. summary statistics (Additional file 1: Table S12).

- 5. Individual well-being questions from MHQ

To investigate the differences in results between the two definitions of well-being (MHQ and GWAS), we analysed the association between genetically instrumented PA and ST time with the three questions that comprise the MHQ well-being score in the UKB. Our findings showed that a 1-SD genetically instrumented higher level of PA was associated with higher levels of general happiness in all individuals (beta: 0.09, 95% CI: 0.03;0.15) and in females only (beta: 0.10, 95% CI: 0.01;0.18), but there was no significant association with happiness with health or meaningful life (Additional file 1: Table S13). Similarly, a 1-SD genetically instrumented higher level of ST was associated with lower levels of general happiness in males only (beta: − 0.19, 95% CI: − 0.37; − 0.01), but there was no significant association with happiness with health or meaningful life (Additional file 1: Table S13).

## Discussion

This MR study provides evidence of a causal bidirectional relationship between objectively measured PA and depression. We confirmed previous findings that higher genetically determined PA associated with lower odds of major depression [19] and provided new evidence that higher PA associated with higher well-being. This study also considered for the first time the role of ST, a distinct phenotype from low PA [16], on mental health outcomes using MR. Higher genetically determined ST was associated with higher odds of anxiety and lower well-being, the latter with the exception of the females only analysis. For the first time, we also highlight bidirectional causal pathways between PA and depression and PA and well-being.

Whilst our UKB only analysis demonstrated no clear evidence of association between PA and lifetime major depression status, this was likely due to a lack of power in UKB as there was robust evidence of an inverse association between PA and lifetime major depression using the PGC summary statistics. This latter result is consistent with previous MR using similar exposure and outcomes [19] as well as with prospective studies showing that those with higher levels of PA had lower odds of depression [3, 6]. Our study goes beyond that of Choi and colleagues by using a MR method specifically designed to account for (a) weak instrument bias, which occurs when instrumenting physical activity and (b) sample overlap, an important source of potential bias when using UKB datasets. We also demonstrated consistent results using our PA and ST instruments in the same depression GWAS as Choi and colleagues, with strong inverse relationships between PA and depression.

Using the individual-level data in UKB we also provided evidence that PA causes lower depression severity. This adds to previous research which has demonstrated that exercise programmes are associated with an amelioration of depressive symptoms [37].

There was no evidence ST was associated with major depression, even when using the larger PGC dataset. Future work should repeat these analyses using the recently published larger GWAS of major depression [30], an analysis we did not perform due to data access constraints.

Our study found that higher genetically determined PA contributed to a higher well-being score, while higher genetically determined ST contributed to a lower well-being score, as defined by the MHQ in UKB. However, we did not observe any significant association between PA, ST and well-being using the subjective well-being definition from the published GWAS [24]. To investigate this difference further, we explored the relationship between higher levels of PA and ST with the individual questions that comprise the UKB well-being score. We found that higher levels of PA were associated with higher levels of general happiness in all individuals and in females only, while higher levels of sedentary time were associated with lower levels of general happiness in males only. There was no significant association between either PA or ST and happiness with health or meaningful life. Our results suggest that the happiness element of subjective well-being is important in the relationship between ST, PA and well-being, but not the meaningful (eudaimonic) or life satisfaction element (cognitive hedonic). This may explain the discrepancy between our UKB results and the GWAS definition of subjective well-being. The published GWAS did not include questions on happiness in their phenotype definition, which our sensitivity analyses suggest is crucial in the PA/ST to well-being relationship. This fits with previous observational literature that shows increasing volumes of PA are associated with higher levels of happiness [38, 39]. Some studies have remained sceptical about the association between PA and happiness, suggesting that the contribution of PA to happiness might be minor compared to other demographic and lifestyle factors, our study provides robust causal evidence for the association between PA and happiness [40].

This study also highlights the importance of ST in mental health and well-being. We add to the evidence base that not only is PA good for well-being, reducing ST will also have beneficial well-being effects [2]. This further highlights that ST is an important construct for health and well-being [14, 41].

We provide evidence for potential causal roles of ST in anxiety. Higher genetically determined ST increased odds of anxiety (PGC summary statistics), but these findings were not consistent when using the UKB definitions of anxiety, although lack of power might explain this discrepancy. Our findings are in agreement with existing evidence of an association between ST and increased anxiety [9, 42].

Our bidirectional analysis provided evidence that higher genetic liability of depression associated with lower PA, but not with higher ST. This differs to the previous MR study [19], but is likely due to using a more recent depression GWAS as our instrument [22] than those used by Choi et al. [43]. The finding of a bidirectional causal association between depression and PA suggests a negative feedback loop where a genetically higher risk of depression causes lower PA which, in turn, increases the risk and severity of depression. No bidirectional associations were observed for anxiety.

This study had many strengths. Firstly, we used objectively measured PA and ST, therefore eliminating the potential effect of self-reported biases. Secondly, we used validated definitions of mental health outcomes [31], this, unlike results from meta-analysis, gives us homogeneity of definitions, an issue that is particularly important in mental health research. Thirdly, we used an MR method accounting for MR biases such as sample overlap and Winner’s curse, which can all affect MR results [32].

We acknowledge several limitations with our study. First, the UKB is not population representative, with over-representation of females and individuals from higher socioeconomic groups [44–46]. Further, our work focused on UKB participants genetically similar to the 1000 genome European ancestry, so our findings might not be generalisable to other ancestries. Second, work by ourselves and others have suggested potential participation biases in the UKB subsets [47] completing the MHQ and physical activity monitoring. However, we have replicated our findings using MDD summary statistics which do not include the UK Biobank, although we acknowledge this will not limit selection bias in our PA and ST metrics. Future work should consider accounting for potential participation biases using recently developed methods [48]. Third, our PA metrics focus on the overall time of PA, there is evidence that the intensity of exercise is also important for mental health, which we were not able to test here. Further the type of PA and ST may also be important in mental health and should be considered in more detail. Fourth, we did not set any specific threshold to account for multiple testing because our mental health phenotypes are correlated, i.e. not truly independent from each other, and therefore corrections such as Bonferroni’s are too conservative. We, instead, report confidence intervals for all our estimates. Fifth, our results are limited to the definitions of mental health available and cannot be extrapolated to different definitions. Finally, whilst we used a range of methods to account for pleiotropy, there was some evidence that our PA instrument predicted lower BMI. We and others have previously demonstrated the importance of BMI in predicting depression status [49, 50], future work should consider and test the potentially mediating effect of BMI on the PA-depression relationship.

## Conclusions

In conclusion, we have highlighted the importance of both PA and ST on a range of mental health outcomes using objectively measured predictors and extensive MR methods for causal inference. Our results are in agreement with other methodological approaches showing the importance of maintaining a high level of PA and reducing ST, for example when desk working. We also highlight the importance of considering bidirectional relationships, with evidence that depression or poor well-being reduces PA. This is important for public health interventions and highlights the need for individuals with depression to be supported to undertake more PA. Our work can be added to the knowledge base suggesting that both PA and ST need to be considered to improve public health.

### Supplementary Information

**Additional file 1.** This file includes the Supplementary methods and Table S1-S13. Tables S1-5 provide the list of SNPs included in the PA, ST, depression, anxiety and wellbeing instrument respectively. Table S6 reports observational associations between PA/ST and mental health. Table S7 summarises any sex differences in our effect estimates. Tables S8-13 represent results from a range of sensitivity analyses. Detailed legends for each table are present in the additional file.

## Data Availability

UKB data are available to any bona fide researcher following application. Including the data on PA and ST: https://www.ukbiobank.ac.uk/enable-your-research/apply-for-access The PA and ST whole cohort summary stats are available here: http://dx.doi.org/10.6084/m9.figshare.24680853 Summary statistics for the mental health metrics are available from the PGC: https://pgc.unc.edu/for-researchers/download-results/ and here http://dx.doi.org/10.6084/m9.figshare.24680853

## Abbreviations

BMI: Body mass index
CIDI-SF: Composite International Diagnostic Interview Short Form
GAD: Generalised anxiety disorder
GWAS: Genome-wide association study
IVW: Inverse variance weighted
MHQ: Mental health questionnaire
MR: Mendelian randomisation
MR-Egger: Egger Mendelian randomisation
PA: Physical activity
PGC: Psychiatric Genomic Consortium
PHQ-9: Patient Health Questionnaire-9
PWM: Penalised weighted median
RCTs: Randomised control trials
SNPs: Single nucleotide polymorphisms
ST: Sedentary time
TDI: Townsend deprivation index
UKB: UK Biobank
WHO: World Health Organisation
WM: Weighted median
