# Supplementary material for: Effects of physical activity and sedentary time on depression, anxiety and well-being: a bidirectional Mendelian randomisation study
Source: BMC Med. 2023 Dec 18;21:501. doi: 10.1186/s12916-023-03211-z (PMC10729457; doi:10.1186/s12916-023-03211-z)
Supplement: Supplementary file 1 — Additional file 1. This file includes the Supplementary methods and Table S1-S13. Tables S1-5 provide the list of SNPs included in the PA, ST, depression, anxiety and wellbeing instrument respectively. Table S6 reports observational associations between PA/ST and mental health. Table S7 summarises any sex differences in our effect estimates. Tables S8-13 represent results from a range of sensitivity analyses. Detailed legends for each table are present in the additional file. [file 12916_2023_3211_MOESM1_ESM.docx]

**Supplementary material:**

Page 2 Supplementary methods

Page 7 Table S1: List of SNPs used for the physical activity instrument. SNPs in known anxiety or depression loci removed in the sensitivity analysis.

Page 11 Table S2: List of SNPs used for the sedentary time instrument. SNPs in known anxiety or depression loci removed in the sensitivity analysis.

Page 13 Table S3: List of SNPs used for the depression instrument from Howard et al.

Page 15 Table S4: List of SNPs used for the anxiety instrument from Otowa et al.

Page 16 Table S5. List of SNPs used for the well-being instrument from Okbay et al.

Page 18 Table S6: Observational associations between sedentary time and mental health outcomes, results are expressed per standard deviation change in sedentary time. All models adjusted for age, sex, body mass index and Townsend deprivation index.

Page 19 Table S7: Results of the Fisher's z analysis comparing betas and standard errors between males and females. Values used for analysis can be found in main manuscript tables 2 to 4.

Page 20 Table S8. Results of the sensitivity analysis excluding SNPs in known depression loci. Results represent odds ratio or betas per standard deviation change in genetically instrumented exposure.

Page 21 Table S9. Results of the inverse weighted (IVW) and pleiotropy robust Egger and Weighted median analysis using physical activity as exposure.

Page 22 Table S10. Results of the inverse weighted (IVW) and pleiotropy robust Egger and Weighted median analysis using sedentary time as exposure.

Page 23 Table S11. Results of the MRLap analysis at different threshold for selecting exposure instrument.

Page 24 Table S12: Results of 2-sample MR analyses using physical activity and sedentary time as exposures and Wray et al., depression summary statistics as the outcome.

Page 25 Table S13. Results of the MRLap analysis of the individual items used in the mental health questionnaire (MHQ) in UK Biobank.

Supplementary Material

We defined 4 continuous variables and 6 binary variables for depression, anxiety and well-being using the well-established and validated Composite International Diagnostic Interview Short Form (CIDI-SF) and the Patient Health Questionnaire-9 (PHQ9), replicating the methods previously described by Davis et al. The 4 continuous variables were: severity of major depression, severity of current depression, severity of anxiety and well-being. The 4 binary variables were: Lifetime major depression, Current depression, Lifetime anxiety disorder rand Current anxiety disorder. All binary continuous variables where derived according to the methods previously described by Davis et al. More detailed description of phenotype derivation can be found in supplementary material from Davis et al. and the detailed R code used can be freely downloaded here: <https://data.mendeley.com/datasets/kv677c2th4/3>

Continuous variables

We created a severity of lifetime depression variable using 8 variables from the CIDI-SF. In each case (unless specifically stated) the options were Yes (score of 1) or No (score of 0), replicating the methods previously described by Davis et al:

• Have you ever had a time in your life when you felt sad, blue, or depressed for two weeks or more in a row? (data field 20446)

• Have you ever had a time in your life lasting two weeks or more when you lost interest in most things like hobbies, work, or activities that usually give you pleasure? (data field 20441)

• Did you feel more tired out or low on energy than is usual for you? (data field 20449)

• Did you gain or lose weight without trying, or did you stay about the same weight? (data field 20536). Here any response other than stayed the same, resulted in adding one to the overall CIDI-SF response variable.

• Did your sleep change? (data field 20532)

• Was that: [re sleep change] Waking too early? (data field 20535)

• Did you have a lot more trouble concentrating than usual? (data field 20435)

• Did you think a lot about death - either your own, someone else's or death in general? (data field 20437)

We created a severity of current depression variable by adding 9 items from the PHQ9, replicating the methods previously described by Davis et al. The items were the answers to “Over the last 2 weeks, how often have you been bothered by any of the following problems (depression symptoms)?”:

• Little interest or pleasure in doing things (data field 20514).

• Feeling bad about yourself or that you are a failure or have let yourself or your family down (data field 20507).

• Feeling down, depressed, or hopeless (data field 20510).

• Trouble concentrating on things, such as reading the newspaper or watching television (data field 20508).

• Trouble falling or staying asleep, or sleeping too much (data field 20517).

• Moving or speaking so slowly that other people could have noticed? Or the opposite - being so fidgety or restless that you have been moving around a lot more than usual (data field 20518).

• Feeling tired or having little energy (data field 20519).

• Thoughts that you would be better off dead or of hurting yourself in some way (data field 20513).

• Poor appetite or overeating (data field 20511).

For each item we scored 1 if the answer was “several days”, 2 if the answers was “more than half the days” and 3 if the answer was “nearly every day”.

We created a severity of anxiety variable by adding 7 items from the mental health questionnaire, replicating the methods previously described by Davis et al. The items were the answers to “Over the last 2 weeks, how often have you been bothered by any of the following problems (anxiety symptoms)?

• Feeling nervous, anxious or on edge (item 20506)

• Not being able to stop or control worrying (item 20509)

• Worrying too much about different things (item 20520)

• Trouble relaxing (item 20515)

• Being so restless that it is hard to sit still (item 20516)

• Becoming easily annoyed or irritable (item 20505)

• Feeling afraid as if something awful might happen (item 20512)

For each item we scored 1 if the answer was “several days”, 2 if the answers was “more than half the days” and 3 if the answer was “nearly every day”.

We created a well-being variable by adding 3 items from the mental health questionnaire, replicating the methods previously described by Davis et al. Two questions provide a euthymic (‘positive emotion’) aspect of well-being and one from the WHO-Quality Of Life (WHOQOL) provides a ‘meaning’ (eudemonic) measure of well-being. The 3 items were:

• In general how happy are you? (data field 20458).

• In general how happy are you with your health? (data field 20459).

For each of these 2 items we scored 1 if the answer was “extremely unhappy”, 2 if “very unhappy”, 3 if “moderately unhappy”, 4 if “moderately happy”, 5 if “very happy” and 6 if “extremely happy”.

• To what extent do you feel your life to be meaningful? (data field 20460).

For this item we scored 1 if the answer was “not at all”, 2 if “a little”, 3 if “a moderate amount”, 4 if “very much” and 5 if “an extreme amount”.

Binary variables

The severity of lifetime depression variable was then utilised to create a binary lifetime major depression variable, where cases were defined based on having at least one core symptom of depression, most or all of the day on most or all days for a two week period, with at least five depressive symptoms that represent a change from usual occurring over the same time-scale, with some or a lot of impairment. Fields and codes used to derive this variable where:

• Persistent sadness (20446) = Yes OR Loss of interest (20441) = Yes AND

• How much of day (20436) = Most of day or All day long AND

• Did you feel this way (20439) = Almost every day or Every day AND

• Impairment (20440) = Somewhat or A lot AND

• Total number of symptoms endorsed >= 5

o Persistent sadness (core) 20446; Loss of interest (core) 20441; Tired or low energy 20449; Gain or loss of weight 20536 = Gain, Loss or Gain and loss; Sleep change 20532; Trouble concentrating 20435; Feeling worthless 20450; Thinking about death 20437

Lifetime major depression controls were defined from the subset who had undertaken the MHQ not endorsing depression or screening positive on PHQ or CIDI. Fields and codes used to derive this variable where:

• NOT (reported diagnosis of depression 20544 or 20002) AND

• NOT symptoms from above case derivation AND

• PHQ score ≤5

This severity of current depression and Lifetime major depression variables were then utilised to create a binary Current depression variable, where cases were defined based on individuals PHQ positive and CIDI positive, Reporting symptoms in the last two weeks that have bothered them. Current depression is indicated by five or more items marked to bother at or above a certain intensity: “more than half of days” for first eight items, “some days” for last item. Fields and codes used to derive this variable where:

• Lifetime major depression cases AND

• Total symptoms endorsed as occurring more than half days (or some or more days for last item) ≥ 5

o Little interest or pleasure in doing things 20514, Feeling down, depressed, or hopeless 20510, Trouble sleeping 20517, Feeling tired 20519, Poor appetite or overeating 20511, Feeling bad about yourself 20507, Trouble concentrating 20508, Moving or speaking slowly or fidgety or restless 20518, Thoughts that you would be better off dead 20513

Current depression controls were defined from the subset who had undertaken the MHQ and who:

• Scored less than 5 in the severity of current depression variable AND

• No self-reported depression and anxiety AND

• No record of depression in hospital episode data AND

• No known use of antidepressants

The severity of anxiety variable was used to derive lifetime generalized anxiety disorder by combining it with further questions from the MHQ. Participants where defined as cases if they reported excessive worrying about a number of issues, occurring most days for six months and difficult to control, with three or more somatic symptoms and functional impairment. Fields and codes used to derive this variable where:

• Worried tense of anxious (20421) = Yes AND

• Duration (20420) >= 6 months or All my life AND

• Most days (20538) = Yes AND

• Excessive: More than most (20425) OR Stronger than most (20542) AND

• Number of issues: More than one thing (20543) OR Different worries (20540) AND

• Difficult to control: Difficult to stop worrying (20541) OR Couldn’t put it out of mind (20539) OR Difficult to control (20537) AND

• Functional impairment: Role interference (20418) = Some or A lot AND

• 3 somatic symptoms out of: Restless. 20426; Keyed up or on edge. 20423; Easily tired. 20429; Having difficulty keeping your mind on what you were doing. 20419; More irritable than usual. 20422; Having tense, sore, or aching muscles. 20417; Often having trouble falling or staying asleep. 20427

Controls for lifetime generalized anxiety disorder were defined as participants who did not meet criteria for lifetime generalized anxiety disorder nor scoring over 5 for severity of anxiety variable.

The severity of anxiety variable was used to derive current generalized anxiety disorder by combining it with further questions from the MHQ. Participants were defined as cases if they had a severity of anxiety score ≥10 and were a case for lifetime generalized anxiety disorder.

Controls for current generalized anxiety disorder were defined as participants who did not meet criteria for current generalized anxiety disorder nor scoring over 10 for severity of anxiety variable.

*Analysis using other MR methods, including pleiotropy robust methods*

Four 2-sample MR methods were performed using a custom pipeline: Inverse-variance weighting (IVW); MR-Egger; Weighted median (WM); Penalised weighted median (PWM). If the genetic variants related to the exposure of interest independently influence the outcome, then horizontal pleiotropy occurs. The IVW method assumes there is either no horizontal pleiotropy under a fixed effects model or, if using a random effects model after detecting heterogeneity amongst the causal estimates, that the strength of the association between the genetic instruments and the exposure is not correlated with the magnitude of the pleiotropic effects (the InSIDE assumption) and that the pleiotropic effects have an average value of zero. An assumption of the weighted median method is that at least 50% of the weight in the analysis stems from variants that are valid instruments. MR-Egger can provide unbiased estimates even when all SNPs violate the exclusion restriction assumption (i.e., they affect the outcome by means other than via the risk factor of interest). However, to use MR-Egger there must be negligible measurement error (NOME) in the genetic instrument and the Instrument Strength Independent of Direct Effect (InSIDE) assumption must be satisfied.

Table S1 List of SNPs used for the physical activity instrument. SNPs in known anxiety or depression loci removed in the sensitivity analysis.

| **SNP** | **CHR** | **BP** | **A1** | **A2** | **A1FREQ** | **BETA** | **SE** | **P** | **Used in PGC depression analysis** | **Used in PGC anxiety analysis** | **Removed in sensitivity analysis** | **Known GWAS hit** | **Approx F** |
| --- | --- | --- | --- | --- | --- | --- | --- | --- | --- | --- | --- | --- | --- |
| rs11810507 | 1 | 9063238 | G | A | 0.847 | 0.030 | 0.006 | 1.30E-06 | Yes | Yes | No | None | 23.5 |
| rs16835316 | 1 | 33691065 | C | G | 0.783 | -0.028 | 0.005 | 1.70E-07 | Yes | Yes | No | None | 27.3 |
| rs141010981 | 1 | 42877403 | G | GGCACAATCTCC | 0.833 | -0.029 | 0.006 | 6.60E-07 | Proxy rs28526231 | Proxy rs28526231 | No | None | 24.5 |
| rs11579616 | 1 | 65304470 | A | C | 0.934 | 0.041 | 0.009 | 4.50E-06 | Yes | Yes | No | None | 21.3 |
| rs140681455 | 1 | 78444764 | C | CGGCCG | 0.874 | 0.035 | 0.007 | 2.90E-07 | Proxy rs34517439 | Proxy rs34517439 | No | Yes, mean corpuscular volume, WHR BMI adjusted | 26.3 |
| 1:111099924 | 1 | 111099924 | CTT | C | 0.792 | -0.027 | 0.005 | 7.00E-07 | Proxy rs72679939 | Proxy rs72679939 | No | None | 24.3 |
| rs791273 | 1 | 147234618 | A | G | 0.748 | -0.025 | 0.005 | 5.70E-07 | Yes | Yes | No | None | 25.1 |
| rs544151060 | 1 | 174272178 | C | CT | 0.761 | 0.024 | 0.005 | 2.90E-06 | Yes | Yes | No | None | 21.8 |
| rs4480415 | 1 | 174800121 | G | A | 0.725 | 0.025 | 0.005 | 1.10E-06 | Yes | Yes | No | None | 23.7 |
| rs757711024 | 1 | 187975472 | TA | T | 0.413 | 0.020 | 0.005 | 7.70E-06 | Yes | No | No | None | 20.5 |
| rs577470297 | 1 | 219808969 | C | CTT | 0.861 | 0.032 | 0.006 | 6.60E-07 | No | Proxy rs113968953 | No | None | 24.8 |
| rs7609303 | 2 | 58101385 | A | T | 0.782 | 0.025 | 0.005 | 4.10E-06 | Yes | Yes | No | None | 21.3 |
| rs55839368 | 2 | 117299962 | G | A | 0.983 | -0.082 | 0.017 | 1.90E-06 | Yes | Yes | No | None | 22.5 |
| rs1220114 | 2 | 158543700 | T | A | 0.743 | 0.024 | 0.005 | 2.10E-06 | Proxy rs10192476 | No | No | None | 22.6 |
| rs259849 | 2 | 180732693 | C | T | 0.607 | -0.020 | 0.005 | 7.10E-06 | Yes | Yes | No | None | 20.0 |
| rs1454556 | 2 | 221156441 | T | C | 0.625 | -0.022 | 0.005 | 8.90E-07 | Yes | Yes | No | None | 23.9 |
| rs9840917 | 3 | 12934141 | A | G | 0.681 | 0.022 | 0.005 | 6.10E-06 | Yes | Yes | No | None | 20.3 |
| rs6775319 | 3 | 18758501 | A | T | 0.270 | 0.024 | 0.005 | 1.80E-06 | Yes | Yes | Yes | Yes, CRP levels | 22.9 |
| rs115803446 | 3 | 82062169 | A | G | 0.950 | 0.048 | 0.010 | 2.30E-06 | Yes | No | No | None | 22.3 |
| rs12488773 | 3 | 86048247 | C | G | 0.608 | -0.023 | 0.005 | 5.10E-07 | Yes | Yes | No | None | 25.1 |
| rs7626095 | 3 | 176320312 | C | T | 0.564 | -0.022 | 0.005 | 1.80E-06 | Yes | Yes | No | None | 23.0 |
| rs7658462 | 4 | 3283422 | C | T | 0.828 | 0.027 | 0.006 | 5.00E-06 | Yes | Yes | Yes | None | 20.9 |
| 4:19566525 | 4 | 19566525 | AAG | A | 0.894 | -0.035 | 0.008 | 7.90E-06 | No | No | No | None | 20.1 |
| rs201118475 | 4 | 49107061 | A | C | 0.248 | -0.025 | 0.005 | 3.30E-06 | No | No | No | None | 21.9 |
| rs72642890 | 4 | 63482431 | G | A | 0.805 | 0.026 | 0.006 | 3.40E-06 | Yes | Yes | No | None | 21.5 |
| 4:83720306 | 4 | 83720306 | CA | C | 0.249 | -0.024 | 0.005 | 5.00E-06 | No | No | No | None | 20.8 |
| rs138061731 | 4 | 121713536 | A | G | 0.983 | -0.077 | 0.017 | 9.70E-06 | Yes | No | No | None | 19.5 |
| rs10857376 | 4 | 166290273 | A | T | 0.799 | -0.026 | 0.006 | 7.70E-06 | No | No | No | None | 19.8 |
| rs10067451 | 5 | 87942506 | G | A | 0.889 | 0.036 | 0.007 | 3.80E-07 | Yes | Yes | No | Yes, photic sneeze reflex | 26.1 |
| rs17135735 | 5 | 112917803 | C | T | 0.929 | 0.043 | 0.009 | 6.20E-07 | Yes | Yes | No | None | 24.6 |
| rs2961762 | 5 | 151924158 | G | T | 0.509 | 0.025 | 0.004 | 1.20E-08 | Yes | Yes | No | None | 32.7 |
| 5:152489987 | 5 | 152489987 | CT | C | 0.477 | 0.021 | 0.005 | 3.70E-06 | No | No | No | None | 21.4 |
| rs4921269 | 5 | 158369472 | A | C | 0.390 | -0.023 | 0.005 | 2.70E-07 | Yes | Yes | No | None | 26.2 |
| rs17065867 | 5 | 165205386 | T | C | 0.962 | 0.051 | 0.011 | 8.10E-06 | Yes | Yes | No | None | 19.9 |
| 6:34708410 | 6 | 34708410 | GGGGAGTAAGTACAAGGTTGCTAGTCT | G | 0.813 | 0.031 | 0.006 | 6.00E-07 | Proxy rs9469899 | Proxy rs9469899 | Yes | None | 25.1 |
| rs546264369 | 6 | 52513418 | G | A | 0.933 | 0.043 | 0.009 | 3.70E-06 | No | No | No | None | 21.2 |
| rs79557053 | 6 | 67500388 | C | T | 0.765 | 0.023 | 0.005 | 8.00E-06 | Yes | Yes | No | None | 19.9 |
| rs144277946 | 6 | 120120386 | C | T | 0.977 | 0.068 | 0.015 | 5.70E-06 | Yes | No | No | None | 20.9 |
| rs180947147 | 6 | 123176726 | G | T | 0.988 | 0.101 | 0.021 | 1.50E-06 | Yes | No | No | None | 23.0 |
| rs17860080 | 7 | 44840638 | G | A | 0.924 | -0.038 | 0.008 | 5.00E-06 | Yes | Yes | No | None | 20.7 |
| rs77392050 | 7 | 64037018 | G | T | 0.922 | -0.036 | 0.008 | 1.10E-05 | Yes | Yes | No | None | 19.6 |
| rs532259022 | 8 | 4856717 | A | C | 0.432 | -0.022 | 0.005 | 1.90E-06 | No | Proxy rs7460368 | No | None | 22.8 |
| rs58097553 | 8 | 63731694 | A | T | 0.785 | -0.024 | 0.005 | 8.40E-06 | No | No | No | None | 19.8 |
| rs117530011 | 8 | 64333246 | G | C | 0.969 | -0.059 | 0.013 | 5.90E-06 | Yes | No | No | None | 20.4 |
| rs74324714 | 8 | 64920807 | A | C | 0.982 | -0.088 | 0.017 | 2.20E-07 | Yes | No | No | None | 27.1 |
| rs142561440 | 8 | 96574531 | C | T | 0.987 | 0.096 | 0.020 | 9.70E-07 | Yes | No | No | None | 23.8 |
| rs10465070 | 9 | 31193534 | C | T | 0.266 | -0.022 | 0.005 | 9.00E-06 | Yes | Yes | Yes | None | 19.7 |
| rs773281 | 9 | 93868693 | G | A | 0.477 | 0.020 | 0.004 | 7.40E-06 | Yes | Yes | No | None | 20.2 |
| rs1268539 | 9 | 128195657 | C | A | 0.582 | -0.027 | 0.004 | 1.60E-09 | Yes | Yes | No | None | 36.4 |
| rs34719019 | 10 | 21885577 | A | T | 0.727 | 0.026 | 0.005 | 1.90E-07 | Yes | Yes | No | None | 27.3 |
| rs181085835 | 10 | 46191657 | C | A | 0.987 | 0.102 | 0.022 | 4.00E-06 | No | No | No | None | 21.2 |
| rs61864473 | 10 | 75561972 | C | T | 0.976 | -0.065 | 0.014 | 6.10E-06 | Yes | No | No | None | 20.2 |
| rs2890087 | 10 | 128845623 | C | T | 0.509 | 0.020 | 0.004 | 3.90E-06 | Yes | Yes | No | None | 21.4 |
| rs78434597 | 11 | 31121145 | G | A | 0.947 | 0.048 | 0.010 | 1.10E-06 | Yes | Yes | No | None | 23.7 |
| rs72892570 | 11 | 31626807 | A | G | 0.948 | 0.049 | 0.010 | 6.20E-07 | Yes | No | No | None | 24.6 |
| rs777529284 | 11 | 57486502 | TATA | T | 0.684 | 0.022 | 0.005 | 4.20E-06 | Proxy rs1785498 | Proxy rs1785498 | Yes | None | 20.9 |
| rs74800845 | 11 | 72380207 | G | A | 0.943 | -0.044 | 0.010 | 3.90E-06 | Yes | Yes | No | None | 21.4 |
| rs10831321 | 11 | 94964343 | A | G | 0.698 | 0.023 | 0.005 | 3.50E-06 | Yes | Yes | No | None | 21.6 |
| rs148193266 | 11 | 104528681 | A | C | 0.957 | -0.059 | 0.011 | 1.20E-07 | Yes | Yes | No | None | 28.3 |
| rs142055384 | 12 | 24304590 | A | G | 0.987 | 0.088 | 0.020 | 7.90E-06 | Yes | No | No | None | 19.9 |
| rs574171779 | 12 | 34301192 | A | AT | 0.447 | -0.022 | 0.005 | 2.40E-06 | Proxy rs11053013 | Proxy rs11053013 | No | None | 22.2 |
| 12:38976006 | 12 | 38976006 | AT | A | 0.502 | -0.021 | 0.004 | 3.30E-06 | Proxy rs11615148 | Proxy rs11615148 | No | None | 21.8 |
| rs78982639 | 12 | 70604120 | G | A | 0.989 | 0.099 | 0.021 | 2.00E-06 | Yes | No | No | None | 22.6 |
| rs35058145 | 12 | 98134238 | G | A | 0.963 | -0.058 | 0.012 | 8.20E-07 | Yes | No | No | None | 24.5 |
| rs9529099 | 13 | 67151771 | C | G | 0.706 | -0.022 | 0.005 | 4.20E-06 | Yes | Yes | Yes | None | 20.6 |
| 14:36992180 | 14 | 36992180 | ATT | A | 0.610 | -0.021 | 0.005 | 6.40E-06 | No | No | No | None | 20.4 |
| rs80336833 | 14 | 70366990 | G | T | 0.955 | -0.049 | 0.011 | 8.40E-06 | Yes | Yes | No | None | 20.0 |
| 14:98658596 | 14 | 98658596 | AT | A | 0.973 | 0.066 | 0.014 | 1.70E-06 | Proxy rs79184814 | No | No | None | 22.9 |
| rs743580 | 15 | 74328116 | A | G | 0.511 | 0.020 | 0.004 | 8.30E-06 | Yes | Yes | No | Yes, insomnia | 20.2 |
| rs560187634 | 15 | 83465785 | A | AC | 0.289 | -0.023 | 0.005 | 4.00E-06 | No | No | No | None | 21.2 |
| 15:90614493 | 15 | 90614493 | TA | T | 0.594 | 0.021 | 0.005 | 8.10E-06 | No | No | No | None | 20.1 |
| rs59065675 | 15 | 91412849 | C | CCAAAGGCA | 0.529 | -0.023 | 0.004 | 5.60E-07 | Proxy rs4932177 | Proxy rs4932177 | No | None | 25.2 |
| rs11852679 | 15 | 92165068 | T | C | 0.678 | 0.027 | 0.005 | 1.20E-07 | Proxy rs8033999 | Yes | No | None | 28.1 |
| rs9938281 | 16 | 49625336 | A | G | 0.475 | -0.020 | 0.004 | 4.90E-06 | Yes | Yes | No | None | 20.9 |
| rs4473187 | 16 | 65190902 | G | A | 0.950 | -0.047 | 0.010 | 2.40E-06 | Yes | Yes | No | None | 22.2 |
| 16:70015522 | 16 | 70015522 | CAA | C | 0.560 | 0.021 | 0.005 | 3.70E-06 | No | Proxy rs62052488 | No | None | 21.2 |
| 16:71673349 | 16 | 71673349 | CT | C | 0.541 | -0.022 | 0.005 | 1.40E-06 | Proxy rs75986475 | Proxy rs75986475 | No | None | 23.1 |
| rs8051200 | 16 | 73468175 | C | T | 0.800 | -0.025 | 0.006 | 4.10E-06 | Yes | Yes | No | None | 21.0 |
| rs72792397 | 16 | 78372637 | G | A | 0.972 | 0.064 | 0.014 | 5.80E-06 | Yes | No | No | None | 20.5 |
| 16:83285681 | 16 | 83285681 | AGTATATAT | A | 0.679 | 0.022 | 0.005 | 5.30E-06 | No | No | No | None | 20.6 |
| rs12149336 | 16 | 89423041 | T | G | 0.488 | 0.020 | 0.004 | 5.80E-06 | Yes | Yes | No | None | 20.5 |
| rs1706730 | 17 | 43671471 | T | C | 0.766 | -0.027 | 0.005 | 2.10E-07 | Proxy rs188710438 | No | No | None | 27.3 |
| rs2696667 | 17 | 44252461 | C | G | 0.848 | -0.038 | 0.007 | 4.90E-08 | Proxy rs80028338 | Proxy rs62055544 | No | None | 30.2 |
| rs553682853 | 17 | 44759766 | G | A | 0.833 | -0.033 | 0.006 | 3.20E-07 | No | No | No | None | 26.2 |
| rs186056728 | 18 | 221643 | C | G | 0.967 | -0.059 | 0.013 | 4.30E-06 | Yes | No | No | None | 21.2 |
| rs1668835 | 18 | 22478952 | T | A | 0.687 | -0.022 | 0.005 | 3.00E-06 | Yes | Yes | No | None | 21.9 |
| rs9965184 | 18 | 40776606 | A | T | 0.654 | -0.024 | 0.005 | 3.50E-07 | Yes | Yes | Yes | Yes, CRP levels | 26.0 |
| rs4806995 | 19 | 4758436 | C | A | 0.644 | 0.022 | 0.005 | 2.70E-06 | Yes | Yes | No | None | 21.9 |
| rs3760731 | 19 | 51375716 | C | T | 0.964 | -0.055 | 0.012 | 7.50E-06 | Yes | No | No | None | 19.8 |
| rs117999688 | 20 | 45963666 | T | C | 0.968 | 0.059 | 0.013 | 4.40E-06 | Yes | No | No | None | 21.0 |
| rs2829752 | 21 | 26830041 | A | G | 0.990 | 0.104 | 0.022 | 1.70E-06 | Yes | No | No | None | 22.5 |
| rs133309 | 22 | 42398606 | G | A | 0.053 | 0.047 | 0.010 | 2.10E-06 | Yes | Yes | No | None | 22.3 |

Table S2: List of SNPs used for the sedentary time instrument. SNPs in known anxiety or depression loci removed in the sensitivity analysis.

| **SNP** | **CHR** | **BP** | **A1** | **A0** | **A1FREQ** | **BETA** | **SE** | **P** | **Used in PGC depression analysis** | **Used in PGC anxiety analysis** | **Removed in sensitivity analysis** | **Known GWAS hit** | **Approx F** |
| --- | --- | --- | --- | --- | --- | --- | --- | --- | --- | --- | --- | --- | --- |
| rs10799238 | 1 | 4568022 | G | A | 0.452 | -0.022 | 0.005 | 1.80E-06 | Yes | Yes | No | None | 22.9 |
| rs76096131 | 1 | 177393818 | T | C | 0.938 | 0.043 | 0.009 | 3.90E-06 | Yes | Yes | No | None | 21.2 |
| rs10916119 | 1 | 227485655 | G | A | 0.825 | 0.029 | 0.006 | 7.10E-06 | No | Yes | No | None | 20.1 |
| rs140515674 | 2 | 103441914 | C | T | 0.985 | -0.085 | 0.019 | 7.10E-06 | Yes | No | No | None | 20.2 |
| rs13385720 | 2 | 135260200 | C | T | 0.919 | 0.037 | 0.008 | 7.00E-06 | Yes | Yes | No | None | 20.1 |
| 2:145796333 | 2 | 145796333 | AT | A | 0.721 | 0.025 | 0.005 | 9.30E-07 | Proxy rs2381684 | Proxy rs2381684 | No | None | 24.0 |
| rs2270334 | 2 | 171675381 | A | G | 0.233 | -0.024 | 0.005 | 7.20E-06 | Yes | Yes | No | None | 20.2 |
| rs369069706 | 3 | 70495848 | C | T | 0.701 | -0.027 | 0.005 | 5.80E-08 | Proxy rs34129269 | Proxy rs34129269 | No | None | 29.0 |
| rs11100789 | 4 | 144337515 | C | T | 0.497 | -0.020 | 0.005 | 6.60E-06 | Yes | Yes | No | None | 20.3 |
| rs2217008 | 4 | 152949744 | C | T | 0.421 | -0.021 | 0.005 | 3.90E-06 | Yes | Yes | No | None | 21.3 |
| 5:6413823 | 5 | 6413823 | ATTAT | A | 0.979 | -0.083 | 0.017 | 1.00E-06 | No | No | No | None | 23.9 |
| 5:88025845 | 5 | 88025845 | CA | C | 0.441 | 0.024 | 0.005 | 1.40E-07 | Proxy rs26579 | Proxy rs26579 | Yes | None | 27.8 |
| rs25981 | 5 | 106822908 | G | C | 0.532 | 0.023 | 0.004 | 2.90E-07 | Yes | Yes | No | None | 26.4 |
| rs6870096 | 5 | 151945811 | C | G | 0.321 | -0.025 | 0.005 | 1.80E-07 | Yes | Yes | No | None | 27.2 |
| rs55924236 | 5 | 177001904 | G | T | 0.878 | 0.033 | 0.007 | 1.90E-06 | Yes | Yes | No | None | 22.7 |
| rs111313891 | 6 | 139032805 | C | A | 0.930 | 0.040 | 0.009 | 7.80E-06 | Proxy rs12529326 | Proxy rs12529326 | No | None | 20.2 |
| 7:64687518 | 7 | 64687518 | AT | A | 0.851 | -0.034 | 0.007 | 5.10E-07 | Proxy rs13223871 | Proxy rs13223871 | No | None | 25.3 |
| rs563423525 | 7 | 67938234 | C | CT | 0.953 | -0.051 | 0.011 | 7.90E-06 | No | No | No | None | 20.1 |
| rs12698833 | 7 | 69466314 | G | A | 0.859 | 0.030 | 0.006 | 3.40E-06 | Yes | Yes | No | None | 21.6 |
| rs2968528 | 7 | 71778063 | G | A | 0.555 | 0.024 | 0.005 | 1.40E-07 | Yes | Yes | No | None | 27.4 |
| rs13229132 | 7 | 89003995 | T | C | 0.987 | -0.118 | 0.026 | 7.60E-06 | No | No | No | None | 20.0 |
| 7:131909139 | 7 | 131909139 | GC | G | 0.986 | 0.098 | 0.021 | 4.60E-06 | No | No | No | None | 21.0 |
| rs553849863 | 8 | 29242547 | A | AT | 0.949 | -0.048 | 0.011 | 5.80E-06 | No | No | No | None | 20.8 |
| rs13265814 | 8 | 107846731 | G | A | 0.985 | -0.084 | 0.019 | 6.20E-06 | Yes | No | No | None | 20.4 |
| rs117849159 | 9 | 22746049 | T | C | 0.988 | -0.105 | 0.022 | 2.60E-06 | Yes | No | No | None | 22.1 |
| rs7855490 | 9 | 129423498 | T | C | 0.785 | -0.024 | 0.006 | 8.60E-06 | Yes | Yes | No | None | 19.6 |
| rs72764982 | 10 | 1623119 | T | G | 0.674 | -0.021 | 0.005 | 9.00E-06 | Yes | Yes | No | None | 19.8 |
| 10:22134285 | 10 | 22134285 | CT | C | 0.318 | 0.023 | 0.005 | 1.60E-06 | Proxy rs11012794 | Proxy rs11012794 | No | None | 22.9 |
| rs11006023 | 10 | 59799718 | C | T | 0.962 | -0.053 | 0.012 | 7.90E-06 | Yes | No | No | None | 20.1 |
| rs149622980 | 10 | 87144254 | G | A | 0.985 | -0.082 | 0.018 | 9.20E-06 | Yes | No | No | None | 19.8 |
| rs11190271 | 10 | 101521777 | A | G | 0.895 | 0.033 | 0.007 | 6.30E-06 | Yes | Yes | No | None | 20.5 |
| rs17076372 | 13 | 22783635 | G | A | 0.972 | -0.062 | 0.014 | 6.50E-06 | Yes | No | No | None | 20.6 |
| rs9548984 | 13 | 40502965 | A | T | 0.539 | 0.021 | 0.004 | 4.10E-06 | Yes | Yes | No | None | 21.1 |
| rs556692971 | 15 | 30110873 | ATT | A | 0.080 | 0.038 | 0.008 | 6.40E-06 | No | No | No | None | 20.5 |
| rs75779711 | 16 | 11859034 | C | A | 0.848 | -0.030 | 0.007 | 9.10E-06 | No | Yes | No | None | 19.7 |
| rs1544812 | 16 | 83390637 | A | T | 0.485 | 0.020 | 0.005 | 8.70E-06 | Yes | Yes | No | None | 19.7 |
| rs3826293 | 17 | 37005017 | C | T | 0.771 | -0.024 | 0.005 | 8.30E-06 | Yes | Yes | No | None | 19.7 |
| rs34855145 | 18 | 27675508 | T | C | 0.898 | 0.033 | 0.007 | 8.70E-06 | Yes | Yes | No | None | 20.0 |
| 18:35346839 | 18 | 35346839 | AT | A | 0.690 | 0.025 | 0.005 | 2.30E-07 | Proxy rs1187219 | Proxy rs1187219 | No | None | 26.9 |
| rs774737943 | 19 | 30924860 | ATGCCTCATATTCTCTCCCACAACCCCCTG | A | 0.943 | -0.045 | 0.010 | 5.00E-06 | No | No |  | None | 20.8 |

Table S3: List of SNPs used for the depression instrument from Howard et al

| SNP | CHR | BP | A1 | A2 | A1FREQ | BETA | SE | P | Known GWAS hit | Approx F |
| --- | --- | --- | --- | --- | --- | --- | --- | --- | --- | --- |
| rs4141983 | 1 | 18122009 | T | C | 0.674 | 0.0264 | 0.0046 | 9.69E-09 | None | 32.9 |
| rs35466861 | 1 | 49803103 | A | G | 0.908 | 0.0461 | 0.0075 | 7.07E-10 | None | 37.8 |
| rs11579246 | 1 | 50559162 | A | G | 0.909 | 0.0491 | 0.0075 | 6.00E-11 | None | 42.9 |
| rs7551758 | 1 | 52274078 | T | G | 0.467 | -0.0283 | 0.0043 | 5.11E-11 | Yes, BMI | 43.3 |
| rs6656912 | 1 | 67083671 | T | C | 0.427 | -0.0252 | 0.0043 | 6.50E-09 | None | 34.3 |
| rs6699744 | 1 | 72825144 | A | T | 0.385 | -0.0392 | 0.0045 | 1.50E-18 | None | 75.9 |
| rs6424508 | 1 | 73507701 | A | G | 0.542 | 0.0294 | 0.0043 | 1.32E-11 | None | 46.7 |
| rs7540134 | 1 | 74077588 | T | C | 0.436 | 0.0255 | 0.0043 | 3.81E-09 | None | 35.2 |
| rs12040241 | 1 | 176000113 | T | C | 0.619 | 0.0259 | 0.0044 | 5.29E-09 | None | 34.6 |
| rs12117045 | 1 | 197638716 | A | G | 0.218 | -0.0295 | 0.0052 | 1.54E-08 | None | 32.2 |
| rs2111592 | 2 | 208049581 | A | G | 0.314 | 0.0263 | 0.0046 | 1.35E-08 | None | 32.7 |
| rs61079605 | 2 | 212621761 | A | T | 0.704 | -0.0265 | 0.0047 | 1.85E-08 | None | 31.8 |
| rs7617480 | 3 | 49210732 | A | C | 0.226 | 0.029 | 0.0051 | 1.68E-08 | Yes, wellbeing, smoking cessation and insomnia | 32.3 |
| rs9851930 | 3 | 49825369 | A | G | 0.175 | 0.0309 | 0.0056 | 4.25E-08 | None | 30.4 |
| rs843812 | 3 | 61255413 | A | G | 0.412 | 0.0248 | 0.0044 | 1.41E-08 | None | 31.8 |
| rs76954012 | 3 | 115977242 | A | T | 0.093 | 0.0412 | 0.0074 | 2.41E-08 | None | 31.0 |
| rs66511648 | 3 | 117515519 | T | C | 0.716 | -0.0297 | 0.0048 | 6.03E-10 | None | 38.3 |
| rs12631196 | 3 | 158171455 | A | G | 0.423 | 0.0241 | 0.0044 | 3.28E-08 | None | 30.0 |
| rs3099439 | 5 | 87545318 | T | C | 0.535 | -0.0241 | 0.0043 | 2.78E-08 | Yes, neuroticism | 31.4 |
| rs30266 | 5 | 103972357 | A | G | 0.327 | 0.0366 | 0.0046 | 1.43E-15 | None | 63.3 |
| rs11135349 | 5 | 164523472 | A | C | 0.467 | -0.029 | 0.0043 | 1.64E-11 | None | 45.5 |
| rs68006638 | 6 | 25710571 | A | G | 0.089 | -0.0428 | 0.0076 | 2.13E-08 | Yes, HDL, neuroticism, wellbeing | 31.7 |
| rs45527431 | 6 | 26599509 | A | G | 0.900 | 0.0545 | 0.0072 | 3.83E-14 | Yes, cognitive function and left handedness | 57.3 |
| rs56114371 | 6 | 27274834 | T | C | 0.100 | -0.0543 | 0.0072 | 4.14E-14 | Yes, triglyceride levels | 56.9 |
| rs200484 | 6 | 27775674 | A | G | 0.874 | 0.0549 | 0.0065 | 2.86E-17 | Yes, haemoglobin measurement | 71.3 |
| rs2232423 | 6 | 28366151 | A | G | 0.894 | 0.062 | 0.007 | 1.14E-18 | Yes, COPD and smoking initiation | 78.4 |
| rs2214123 | 6 | 67000001 | A | G | 0.353 | 0.0261 | 0.0045 | 8.56E-09 | None | 33.6 |
| rs2876520 | 6 | 142996618 | C | G | 0.531 | -0.026 | 0.0043 | 2.24E-09 | None | 36.6 |
| rs9364755 | 6 | 165117329 | A | G | 0.774 | -0.0283 | 0.0051 | 3.49E-08 | None | 30.8 |
| rs10235664 | 7 | 2086814 | T | C | 0.747 | 0.027 | 0.0049 | 4.68E-08 | Yes, PTSD | 30.4 |
| rs3807865 | 7 | 12250402 | A | G | 0.411 | 0.031 | 0.0044 | 1.09E-12 | Yes, aspartate aminotransferase levels | 49.6 |
| rs59082935 | 7 | 38724868 | T | C | 0.134 | 0.0363 | 0.0066 | 3.07E-08 | None | 30.3 |
| rs2247523 | 7 | 82454404 | C | G | 0.530 | -0.0243 | 0.0043 | 1.71E-08 | None | 31.9 |
| rs4730387 | 7 | 109100414 | A | T | 0.466 | 0.0238 | 0.0043 | 4.12E-08 | None | 30.6 |
| rs150346963 | 7 | 117625599 | T | C | 0.412 | 0.0283 | 0.0044 | 1.16E-10 | None | 41.4 |
| rs1931388 | 9 | 11203149 | A | G | 0.596 | 0.0295 | 0.0044 | 1.68E-11 | None | 45.0 |
| rs55833444 | 9 | 11703866 | A | C | 0.223 | -0.0292 | 0.0052 | 1.68E-08 | None | 31.5 |
| rs263645 | 9 | 17016503 | A | T | 0.541 | 0.0251 | 0.0043 | 6.36E-09 | None | 34.1 |
| rs59283172 | 9 | 25232978 | A | G | 0.108 | -0.039 | 0.007 | 2.41E-08 | None | 31.0 |
| rs62535714 | 9 | 37182655 | A | G | 0.164 | 0.0339 | 0.0058 | 4.69E-09 | None | 34.2 |
| rs2418449 | 9 | 119731359 | T | C | 0.719 | 0.0281 | 0.0048 | 4.25E-09 | None | 34.3 |
| rs1021363 | 10 | 106610839 | A | G | 0.357 | 0.03 | 0.0045 | 2.29E-11 | Yes, GORD | 44.4 |
| rs198457 | 11 | 61471678 | T | C | 0.189 | -0.0315 | 0.0056 | 1.90E-08 | Yes, neuroticism, wellbeing, phospholipid levels | 31.6 |
| rs10501696 | 11 | 88748162 | A | G | 0.505 | 0.0295 | 0.0044 | 2.89E-11 | None | 45.0 |
| rs4936275 | 11 | 113365084 | T | C | 0.622 | 0.0278 | 0.0044 | 3.35E-10 | None | 39.9 |
| rs11612312 | 12 | 52349088 | T | C | 0.797 | -0.0309 | 0.0054 | 8.03E-09 | None | 32.7 |
| rs9529314 | 13 | 31799646 | A | G | 0.203 | -0.0335 | 0.0053 | 3.72E-10 | None | 40.0 |
| rs9536381 | 13 | 53860655 | T | C | 0.326 | 0.0255 | 0.0046 | 2.62E-08 | None | 30.7 |
| rs508502 | 13 | 80921519 | T | C | 0.299 | -0.0264 | 0.0048 | 3.56E-08 | None | 30.3 |
| rs1950829 | 14 | 42097937 | A | G | 0.483 | 0.0297 | 0.0043 | 4.74E-12 | None | 47.7 |
| rs7152906 | 14 | 75125540 | T | C | 0.480 | -0.0258 | 0.0043 | 1.87E-09 | None | 36.0 |
| rs754287 | 14 | 103997525 | A | T | 0.366 | -0.0289 | 0.0045 | 1.31E-10 | None | 41.2 |
| rs28541419 | 15 | 88945878 | C | G | 0.769 | 0.0292 | 0.0052 | 1.76E-08 | None | 31.5 |
| rs7200826 | 16 | 13066833 | T | C | 0.257 | 0.0268 | 0.0049 | 5.49E-08 | Yes, neuroticism | 29.9 |
| rs12919291 | 16 | 13800430 | C | G | 0.188 | 0.0327 | 0.0055 | 3.09E-09 | None | 35.3 |
| rs12967855 | 18 | 35138245 | A | G | 0.330 | 0.0294 | 0.0046 | 1.50E-10 | Yes, education, household income | 40.8 |
| rs17410557 | 18 | 50776391 | T | C | 0.608 | -0.0267 | 0.0044 | 1.42E-09 | Yes, multisite pain | 36.8 |
| rs8093506 | 18 | 52474170 | A | G | 0.765 | 0.0291 | 0.0051 | 1.33E-08 | None | 32.6 |
| rs12967143 | 18 | 53099012 | C | G | 0.701 | -0.0345 | 0.0047 | 2.53E-13 | Yes, insomnia, cigarettes per day | 53.9 |
| rs7241572 | 18 | 77580712 | A | G | 0.205 | 0.0323 | 0.0054 | 2.43E-09 | None | 35.8 |
| rs13037326 | 20 | 44692598 | T | C | 0.260 | 0.031 | 0.0049 | 2.40E-10 | None | 40.0 |

Table S4: List of SNPs used for the anxiety instrument from Otowa et al.

| SNP | CHR | BP | A1 | A2 | A1FREQ | BETA | SE | P | Known GWAS hit | Approximated F |
| --- | --- | --- | --- | --- | --- | --- | --- | --- | --- | --- |
| rs356407 | 2 | 180616609 | A | C | 0.3046 | 0.139 | 0.031 | 7.33E-06 | None | 20.1 |
| rs1709393 | 3 | 101699154 | T | C | 0.5793 | -0.1509 | 0.0267 | 1.65E-08 | None | 31.9 |
| rs6774302 | 3 | 177866284 | A | G | 0.1176 | -0.2548 | 0.0571 | 8.19E-06 | None | 19.9 |
| rs6601080 | 5 | 179511043 | A | G | 0.6654 | -0.1282 | 0.0282 | 5.33E-06 | Yes, insomnia | 20.7 |
| rs6887011 | 5 | 124686839 | A | C | 0.3719 | 0.1234 | 0.0276 | 7.78E-06 | None | 20.0 |
| rs72817576 | 5 | 164586884 | A | T | 0.0551 | -0.4851 | 0.1084 | 7.62E-06 | None | 20.0 |
| rs2146346 | 6 | 11167111 | A | G | 0.5869 | 0.1441 | 0.0298 | 1.38E-06 | None | 23.4 |
| rs2645117 | 6 | 37954153 | T | G | 0.4834 | 0.1473 | 0.0321 | 4.51E-06 | None | 21.1 |
| rs2753188 | 6 | 47941339 | A | G | 0.7268 | 0.1603 | 0.0332 | 1.42E-06 | None | 23.3 |
| rs112205280 | 7 | 153760066 | A | G | 0.5937 | 0.3532 | 0.0719 | 9.03E-07 | None | 24.1 |
| rs10814991 | 9 | 4495254 | T | C | 0.3916 | -0.1664 | 0.0368 | 6.23E-06 | None | 20.4 |
| rs10766527 | 11 | 19100736 | A | G | 0.8723 | 0.1998 | 0.0452 | 9.91E-06 | None | 19.5 |
| rs58990403 | 11 | 116509339 | A | G | 0.792 | -0.1851 | 0.0391 | 2.16E-06 | None | 22.4 |
| rs56070849 | 13 | 108073449 | T | C | 0.8822 | 0.2104 | 0.0463 | 5.46E-06 | None | 20.7 |
| rs16963010 | 15 | 50170441 | T | C | 0.1635 | 0.1763 | 0.039 | 6.01E-06 | None | 20.4 |
| rs2124973 | 15 | 37729549 | T | C | 0.94 | -0.4557 | 0.0999 | 5.05E-06 | None | 20.8 |
| rs28373923 | 16 | 88815473 | A | G | 0.0675 | 0.4193 | 0.0915 | 4.56E-06 | None | 21.0 |
| rs2740360 | 17 | 629309 | T | C | 0.4743 | 0.1696 | 0.0334 | 3.81E-07 | None | 25.8 |
| rs874707 | 17 | 71425494 | T | C | 0.483 | -0.1412 | 0.0317 | 8.30E-06 | None | 19.8 |
| rs739315 | 22 | 25617602 | A | G | 0.5688 | -0.1537 | 0.0329 | 3.03E-06 | None | 21.8 |

Table S5. List of SNPs used for the well-being instrument from Okbay et al

| SNP | CHR | BP | A1 | A2 | A1FREQ | BETA | SE | P | Known GWAS hit | Approximated F |
| --- | --- | --- | --- | --- | --- | --- | --- | --- | --- | --- |
| rs6587766 | 1 | 57708088 | T | C | 0.041 | 0.047 | 0.009 | 2.52E-07 | None | 27.3 |
| rs12143280 | 1 | 211547522 | T | C | 0.944 | 0.038 | 0.008 | 6.46E-07 | None | 22.6 |
| rs4666465 | 2 | 19338691 | T | C | 0.338 | 0.014 | 0.003 | 6.34E-05 | Yes, glaucoma diagnostic measure | 21.8 |
| rs7584895 | 2 | 29229016 | T | C | 0.519 | -0.014 | 0.003 | 1.15E-05 | None | 21.8 |
| rs7609322 | 2 | 47805801 | C | G | 0.511 | 0.014 | 0.003 | 6.09E-05 | None | 21.8 |
| rs6758268 | 2 | 134583257 | A | G | 0.138 | -0.018 | 0.004 | 4.26E-05 | None | 20.3 |
| rs13387164 | 2 | 154144551 | A | G | 0.091 | -0.023 | 0.005 | 5.98E-06 | None | 21.2 |
| rs11691770 | 2 | 168633442 | T | G | 0.039 | 0.037 | 0.008 | 8.52E-06 | None | 21.4 |
| rs905456 | 3 | 30746255 | A | G | 0.429 | -0.014 | 0.003 | 1.48E-05 | None | 21.8 |
| rs4589952 | 3 | 95702105 | T | C | 0.155 | -0.021 | 0.004 | 1.55E-06 | None | 27.6 |
| rs17331012 | 4 | 12144655 | A | G | 0.129 | -0.023 | 0.005 | 1.10E-06 | Yes, height | 21.2 |
| rs6813656 | 4 | 82380368 | T | C | 0.968 | -0.045 | 0.010 | 7.74E-06 | None | 20.3 |
| rs6830177 | 4 | 140976736 | A | T | 0.369 | -0.014 | 0.003 | 3.08E-05 | None | 21.8 |
| rs11731303 | 4 | 149999533 | C | G | 0.478 | -0.014 | 0.003 | 5.52E-05 | None | 21.8 |
| rs2597455 | 4 | 163307671 | T | G | 0.623 | -0.014 | 0.003 | 1.78E-05 | None | 21.8 |
| rs925894 | 5 | 124723432 | T | C | 0.399 | -0.014 | 0.003 | 2.31E-05 | None | 21.8 |
| rs6913660 | 6 | 27091425 | A | C | 0.170 | 0.018 | 0.004 | 2.09E-05 | None | 20.3 |
| rs17693963 | 6 | 27710165 | A | C | 0.897 | -0.026 | 0.005 | 9.25E-07 | Yes, schizophrenia | 27.0 |
| rs7752448 | 6 | 28301099 | A | G | 0.886 | -0.025 | 0.005 | 1.46E-06 | Yes, lung function, GORD | 25.0 |
| rs11755393 | 6 | 34824636 | A | G | 0.647 | -0.014 | 0.003 | 5.24E-05 | Yes, BMI | 21.8 |
| rs9388300 | 6 | 124416847 | T | C | 0.845 | -0.018 | 0.004 | 6.55E-05 | None | 20.3 |
| rs13235506 | 7 | 53785701 | T | G | 0.955 | -0.034 | 0.007 | 1.96E-06 | None | 23.6 |
| rs2865116 | 7 | 69042724 | A | G | 0.688 | 0.015 | 0.003 | 1.30E-05 | None | 25.0 |
| rs2058382 | 7 | 69579024 | A | G | 0.590 | -0.015 | 0.003 | 6.12E-06 | None | 25.0 |
| rs258677 | 7 | 81733428 | T | G | 0.375 | 0.017 | 0.003 | 9.46E-08 | None | 32.1 |
| rs1075737 | 7 | 103071193 | A | G | 0.185 | -0.019 | 0.004 | 8.11E-06 | None | 22.6 |
| rs4570163 | 8 | 123451096 | T | C | 0.702 | -0.014 | 0.003 | 1.54E-05 | None | 21.8 |
| rs1556477 | 9 | 20735284 | A | G | 0.634 | 0.014 | 0.003 | 3.42E-05 | None | 21.8 |
| rs2017279 | 10 | 3534725 | A | G | 0.679 | 0.016 | 0.003 | 3.15E-06 | None | 28.4 |
| rs1361360 | 10 | 20795797 | T | C | 0.401 | 0.014 | 0.003 | 3.30E-05 | None | 21.8 |
| rs17627437 | 10 | 36681567 | T | C | 0.841 | -0.018 | 0.004 | 3.64E-05 | None | 20.3 |
| rs7072297 | 10 | 44462360 | C | G | 0.843 | 0.018 | 0.004 | 3.55E-05 | None | 20.3 |
| rs6479789 | 10 | 63947969 | T | C | 0.832 | 0.018 | 0.004 | 1.92E-05 | None | 20.3 |
| rs1530584 | 11 | 3166068 | A | G | 0.418 | 0.014 | 0.003 | 6.24E-05 | None | 21.8 |
| rs903834 | 11 | 10814116 | A | G | 0.407 | 0.015 | 0.003 | 1.17E-05 | None | 25.0 |
| rs10769190 | 11 | 46151490 | T | C | 0.198 | 0.018 | 0.004 | 2.19E-06 | Yes, height | 20.3 |
| rs3816605 | 11 | 47857253 | T | C | 0.535 | 0.014 | 0.003 | 1.46E-05 | Yes, BMI and Type 2 Diabetes | 21.8 |
| rs1384753 | 11 | 83655564 | T | C | 0.836 | -0.018 | 0.004 | 7.79E-05 | None | 20.3 |
| rs976337 | 11 | 95011645 | T | C | 0.597 | 0.014 | 0.003 | 5.27E-06 | None | 21.8 |
| rs7939430 | 11 | 134631823 | A | G | 0.149 | -0.019 | 0.004 | 3.40E-05 | None | 22.6 |
| rs11612312 | 12 | 52349088 | T | C | 0.806 | 0.019 | 0.004 | 1.37E-06 | None | 22.6 |
| rs12298541 | 12 | 66306441 | A | C | 0.356 | 0.016 | 0.003 | 2.28E-06 | None | 28.4 |
| rs6581971 | 12 | 71289408 | T | C | 0.832 | -0.019 | 0.004 | 1.33E-05 | None | 22.6 |
| rs4842284 | 12 | 80823060 | T | C | 0.080 | 0.031 | 0.006 | 8.01E-07 | None | 26.7 |
| rs11043207 | 12 | 122268696 | C | G | 0.797 | 0.018 | 0.004 | 3.84E-05 | Yes, mean platelet volume, mean corpuscular volume | 20.3 |
| rs7149000 | 14 | 41897318 | A | G | 0.459 | -0.014 | 0.003 | 4.77E-06 | None | 21.8 |
| rs11161293 | 15 | 26602423 | T | G | 0.868 | -0.018 | 0.004 | 6.23E-05 | None | 20.3 |
| rs419055 | 15 | 47676519 | A | T | 0.705 | 0.014 | 0.003 | 5.30E-05 | None | 21.8 |
| rs11073619 | 15 | 85188839 | T | C | 0.105 | 0.025 | 0.005 | 3.42E-06 | None | 25.0 |
| rs11644362 | 16 | 12994097 | T | C | 0.459 | 0.014 | 0.003 | 2.06E-05 | Yes, neuroticism | 21.8 |
| rs7189864 | 16 | 73633936 | C | G | 0.313 | -0.014 | 0.003 | 2.52E-05 | Yes, photic sneeze reflex | 21.8 |
| rs4792196 | 17 | 11820388 | A | T | 0.188 | 0.018 | 0.004 | 4.18E-05 | None | 20.3 |
| rs10459946 | 17 | 65613027 | A | G | 0.621 | -0.014 | 0.003 | 7.02E-05 | None | 21.8 |
| rs12943580 | 17 | 72166219 | A | G | 0.397 | -0.014 | 0.003 | 4.77E-05 | None | 21.8 |
| rs8071392 | 17 | 79091103 | T | G | 0.847 | -0.018 | 0.004 | 7.18E-05 | None | 20.3 |
| rs2120884 | 18 | 49749024 | A | C | 0.179 | 0.018 | 0.004 | 7.10E-05 | None | 20.3 |
| rs7239568 | 18 | 51964773 | A | C | 0.688 | -0.016 | 0.003 | 4.58E-06 | None | 28.4 |
| rs7239776 | 18 | 52578599 | A | T | 0.530 | -0.016 | 0.003 | 3.31E-07 | None | 28.4 |
| rs12460988 | 19 | 31162121 | T | C | 0.573 | -0.014 | 0.003 | 1.67E-05 | None | 21.8 |
| rs11671324 | 19 | 57515403 | T | C | 0.912 | 0.027 | 0.006 | 3.54E-05 | None | 20.3 |
| rs6073597 | 20 | 43629512 | T | C | 0.552 | 0.014 | 0.003 | 7.96E-06 | None | 21.8 |
| rs348276 | 20 | 47786190 | A | G | 0.321 | -0.019 | 0.003 | 3.53E-08 | Yes, cognitive ability | 40.1 |
| rs6089930 | 20 | 61359001 | A | G | 0.457 | -0.014 | 0.003 | 1.32E-05 | None | 21.8 |
| rs12152057 | 21 | 25056215 | A | C | 0.674 | 0.015 | 0.003 | 1.14E-05 | None | 25.0 |

Table S6: Observational associations between sedentary time and mental health outcomes, results are expressed per standard deviation change in sedentary time. All models adjusted for age, sex, body mass index and Townsend deprivation index.

| **exposure** | **outcome** | **strata** | **Odds Ratio (95%CI)** | **P** |  | **exposure** | **outcome** | **strata** | **Odds Ratio (95%CI)** | **P** |
| --- | --- | --- | --- | --- | --- | --- | --- | --- | --- | --- |
| Physical activity | Current depression | all | 0.704 (0.658;0.752) | < 2.00E-16 |  | Sedentary time | Current depression | all | 0.992 (0.929;1.059) | 8.04E-01 |
| Physical activity |  | females | 0.702 (0.645;0.764) | < 2.00E-16 |  | Sedentary time |  | females | 0.99 (0.911;1.076) | 8.13E-01 |
| Physical activity |  | males | 0.707 (0.633;0.791) | 1.19E-09 |  | Sedentary time |  | males | 0.992 (0.892;1.104) | 8.86E-01 |
| Physical activity | Lifetime major depression | all | 0.883 (0.865;0.902) | < 2.00E-16 |  | Sedentary time | Lifetime major depression | all | 1.019 (0.998;1.041) | 7.43E-02 |
| Physical activity |  | females | 0.866 (0.843;0.889) | < 2.00E-16 |  | Sedentary time |  | females | 1.049 (1.022;1.076) | 3.80E-04 |
| Physical activity |  | males | 0.915 (0.883;0.947) | 7.64E-07 |  | Sedentary time |  | males | 0.972 (0.939;1.006) | 1.01E-01 |
| Physical activity | Current anxiety disorder | all | 0.820 (0.756;0.889) | 1.71E-06 |  | Sedentary time | Current anxiety disorder | all | 0.958 (0.883;1.038) | 2.94E-01 |
| Physical activity |  | females | 0.800 (0.725;0.883) | 8.61E-06 |  | Sedentary time |  | females | 0.992 (0.898;1.095) | 8.73E-01 |
| Physical activity |  | males | 0.863 (0.747;0.997) | 4.62E-02 |  | Sedentary time |  | males | 0.893 (0.778;1.024) | 1.06E-01 |
| Physical activity | Lifetime anxiety disorder | all | 0.868 (0.832;0.906) | 7.36E-11 |  | Sedentary time | Lifetime anxiety disorder | all | 0.958 (0.919;0.999) | 4.45E-02 |
| Physical activity |  | females | 0.848 (0.804;0.894) | 1.13E-09 |  | Sedentary time |  | females | 0.974 (0.923;1.027) | 3.23E-01 |
| Physical activity |  | males | 0.909 (0.847;0.976) | 8.18E-03 |  | Sedentary time |  | males | 0.933 (0.872;0.999) | 4.77E-02 |
| **exposure** | **outcome** | **strata** | **Beta (95%CI)** | **P** |  | **exposure** | **outcome** | **strata** | **Beta (95%CI)** | **P** |
| Physical activity | Well-being | all | 0.269 (0.251;0.286) | < 2.00E-16 |  | Sedentary time | Well-being | all | -0.109 (-0.126;-0.092) | < 2.00E-16 |
| Physical activity |  | females | 0.284 (0.261;0.308) | < 2.00E-16 |  | Sedentary time |  | females | -0.120 (-0.143;-0.096) | < 2.00E-16 |
| Physical activity |  | males | 0.254 (0.228;0.280) | < 2.00E-16 |  | Sedentary time |  | males | -0.097 (-0.122;-0.072) | 2.90E-14 |
| Physical activity | Severity of major depression | all | -0.147 (-0.172;-0.122) | < 2.00E-16 |  | Sedentary time | Severity of major depression | all | 0.000 (-0.024;0.025) | 9.72E-01 |
| Physical activity |  | females | -0.188 (-0.224;-0.153) | < 2.00E-16 |  | Sedentary time |  | females | 0.042 (0.007;0.077) | 1.78E-02 |
| Physical activity |  | males | -0.099 (-0.134;-0.064) | 3.47E-08 |  | Sedentary time |  | males | -0.047 (-0.081;-0.013) | 6.96E-03 |
| Physical activity | Severity of current depression | all | -0.361 (-0.392;-0.330) | < 2.00E-16 |  | Sedentary time | Severity of current depression | all | 0.092 (0.062;0.122) | 2.92E-09 |
| Physical activity |  | females | -0.404 (-0.448;-0.361) | < 2.00E-16 |  | Sedentary time |  | females | 0.118 (0.075;0.161) | 7.91E-08 |
| Physical activity |  | males | -0.308 (-0.352;-0.265) | < 2.00E-16 |  | Sedentary time |  | males | 0.062 (0.020;0.104) | 4.19E-03 |
| Physical activity | Severity of anxiety | all | -0.157 (-0.186;-0.128) | < 2.00E-16 |  | Sedentary time | Severity of anxiety | all | -0.017 (-0.045;0.011) | 2.31E-01 |
| Physical activity |  | females | -0.185 (-0.227;-0.143) | < 2.00E-16 |  | Sedentary time |  | females | -0.031 (-0.072;0.010) | 1.44E-01 |
| Physical activity |  | males | -0.123 (-0.162;-0.084) | 4.35E-10 |  | Sedentary time |  | males | -0.002 (-0.040;0.035) | 8.97E-01 |

All models adjusted for age, sex, body mass index and Townsend deprivation index.

Table S7: Results of the Fisher's z analysis comparing betas and standard errors between males and females. Values used for analysis can be found in main manuscript tables 2 to 4.

| **exposure** | **outcome** | **Fisher's Z between males and females** | **p** |
| --- | --- | --- | --- |
| Physical activity | Current depression | -0.391 | 6.95E-01 |
|  | Lifetime major depression | -0.359 | 7.20E-01 |
|  | Current anxiety disorder | -0.349 | 7.27E-01 |
|  | Lifetime anxiety disorder | 0.085 | 9.32E-01 |
|  |  |  |  |
| Physical activity | Well-being | -0.447 | 6.55E-01 |
|  | Severity of major depression | 0.624 | 5.33E-01 |
|  | Severity of current depression | 0.899 | 3.69E-01 |
|  | Severity of anxiety | 1.821 | 6.87E-02 |
|  |  |  |  |
| Sedentary time | Current depression | -0.121 | 9.04E-01 |
|  | Lifetime major depression | -0.446 | 6.55E-01 |
|  | Current anxiety disorder | 1.525 | 1.27E-01 |
|  | Lifetime anxiety disorder | 0.262 | 7.93E-01 |
|  |  |  |  |
| Sedentary time | Well-being | -0.693 | 4.88E-01 |
|  | Severity of major depression | 0.674 | 5.01E-01 |
|  | Severity of current depression | -0.243 | 8.08E-01 |
|  | Severity of anxiety | -0.507 | 6.12E-01 |
| **Exposure** | **Outcome** | **Fisher's Z between males and females** | **p** |
| PGC Depression | Overall physical activity | 2.531 | 1.14E-02 |
| PG Depression | Sedentary time | -1.753 | 7.97E-02 |
|  |  |  |  |
| Anxiety | Overall physical activity | -0.251 | 8.02E-01 |
| Anxiety | Sedentary time | 0.408 | 6.83E-01 |
|  |  |  |  |
| Well-being GWAS | Overall physical activity | 0.557 | 5.77E-01 |
| Wellbeing GWAS | Sedentary time | 0.283 | 7.77E-01 |
|  |  |  |  |
| Well-being from UKB | Overall physical activity | -0.665 | 5.06E-01 |
| Well-being from UKB | Sedentary time | 0.546 | 5.85E-01 |

Table S8: Results of the sensitivity analysis excluding SNPs in known depression loci. Results represent odds ratio or betas per standard deviation change in genetically instrumented exposure.

| **exposure** | **outcome** | **strata** | **Beta (SE)** | **P** |
| --- | --- | --- | --- | --- |
| Physical activity | Well-being | all | 0.098 (0.029;0.166) | 5.47E-03 |
|  |  | females | 0.109 (0.048;0.203) | 2.37E-02 |
|  |  | males | 0.077 (-0.017;0.170) | 1.07E-01 |
|  |  |  |  |  |
| Physical activity | Severity of current depression | all | -0.062 (-0.149;-0.017) | 1.35E-02 |
|  |  | females | -0.107 (-0.194;-0.096) | 1.69E-02 |
|  |  | males | -0.049 (-0.128;0.030) | 2.26E-01 |
| **exposure** | **outcome** | **strata** | **OR (95%CI** | **P** |
| Physical activity | PGC-Depression | all | 0.940 (0.894;0.989) | 1.64E-02 |
| **exposure** | **outcome** | **strata** | **Beta (SE)** | **P** |
| Sedentary time | Well-being | all | -0.189 (-0.324;-0.030) | 1.93E-02 |
|  |  | females | -0.170 (-0.354;0.013) | 6.91E-02 |
|  |  | males | -0.213 (-0.436;0.009) | 6.02E-02 |
|  |  |  |  |  |
| Sedentary | PGC-Anxiety | all | 2.250 (1.107;4.575) | 2.49E-02 |
|  |  |  |  |  |
| PGC depression | Physical activity | All | -0.251 (-0.418;-0.084) | 3.24E-03 |
|  |  | females | -0.451 (-0.672;-0.230) | 6.85E-05 |
|  |  | males | -0.076 (-0.267;-0.114) | 4.33E-01 |
|  |  |  |  |  |
|  |  | all | 0.110 (0.026;0.194) | 1.01E-02 |
| Well-being from UKB | Overall physical activity | females | 0.140 (0.019;0.261) | 2.24E-02 |
|  |  | males | 0.082 (-0.014;0.179) | 9.25E-02 |

Table S9: Results of the inverse weighted (IVW) and pleiotropy robust Egger and Weighted median analysis using physical activity as exposure.

|  |  |  | **IVW** |  |  | **Egger** |  |  | **Weighted Median** |  |
| --- | --- | --- | --- | --- | --- | --- | --- | --- | --- | --- |
| **exposure** | **outcome** | **strata** | **OR (95%CI)** | **P** |  | **OR (95%CI)** | **P** |  | **OR (95%CI)** | **P** |
| Physical activity | Current depression | all | 0.841 (0.642;1.102) | 2.12E-01 |  | 0.837 (0.639;1.097) | 2.00E-01 |  | 0.750 (0.509;1.105) | 1.46E-01 |
| Physical activity |  | females | 0.864 (0.616;1.213) | 4.01E-01 |  | 0.863 (0.614;1.211) | 3.95E-01 |  | 0.821 (0.505;1.336) | 4.28E-01 |
| Physical activity |  | males | 0.788 (0.505;1.229) | 2.96E-01 |  | 0.781 (0.500;1.218) | 2.79E-01 |  | 0.957 (0.512;1.786) | 8.89E-01 |
| Physical activity | Lifetime major depression | all | 0.975 (0.877;1.084) | 6.41E-01 |  | 0.974 (0.876;1.083) | 6.28E-01 |  | 0.902 (0.794;1.024) | 1.12E-01 |
| Physical activity |  | females | 0.989 (0.876;1.116) | 8.52E-01 |  | 0.989 (0.876;1.117) | 8.57E-01 |  | 0.858 (0.727;1.012) | 6.85E-02 |
| Physical activity |  | males | 0.952 (0.802;1.129) | 5.71E-01 |  | 0.948 (0.800;1.123) | 5.38E-01 |  | 1.039 (0.838;1.290) | 7.26E-01 |
| Physical activity | Current anxiety disorder | all | 0.727 (0.511;1.034) | 7.94E-02 |  | 0.722 (0.508;1.026) | 7.28E-02 |  | 0.792 (0.495;1.267) | 3.31E-01 |
| Physical activity |  | females | 0.754 (0.504;1.128) | 1.73E-01 |  | 0.750 (0.501;1.122) | 1.65E-01 |  | 0.648 (0.372;1.129) | 1.26E-01 |
| Physical activity |  | males | 0.675 (0.382;1.193) | 1.80E-01 |  | 0.670 (0.379;1.184) | 1.71E-01 |  | 0.613 (0.281;1.337) | 2.19E-01 |
| Physical activity | Lifetime anxiety disorder | all | 0.948 (0.796;1.127) | 5.45E-01 |  | 0.947 (0.795;1.128) | 5.46E-01 |  | 0.840 (0.654;1.078) | 1.70E-01 |
| Physical activity |  | females | 0.916 (0.735;1.141) | 4.34E-01 |  | 0.917 (0.735;1.144) | 4.46E-01 |  | 0.906 (0.665;1.234) | 5.30E-01 |
| Physical activity |  | males | 0.948 (0.714;1.258) | 7.12E-01 |  | 0.943 (0.710;1.251) | 6.83E-01 |  | 0.833 (0.549;1.262) | 3.88E-01 |
| **exposure** | **outcome** | **strata** |  |  |  |  |  |  |  |  |
| Physical activity | Well-being | all | 0.200 (0.113;0.287) | 2.09E-05 |  | 0.201 (0.113;0.288) | 2.19E-05 |  | 0.173 (0.064;0.283) | 1.89E-03 |
| Physical activity |  | females | 0.216 (0.099;0.333) | 4.89E-04 |  | 0.216 (0.098;0.334) | 5.42E-04 |  | 0.092 (-0.055;0.239) | 2.18E-01 |
| Physical activity |  | males | 0.177 (0.067;0.287) | 2.16E-03 |  | 0.179 (0.069;0.289) | 1.95E-03 |  | 0.146 (-0.014;0.306) | 7.37E-02 |
| Physical activity | Severity of major depression | all | -0.052 (-0.209;0.105) | 5.16E-01 |  | -0.054 (-0.211;0.103) | 5.01E-01 |  | -0.105 (-0.265;0.056) | 2.01E-01 |
| Physical activity |  | females | -0.090 (-0.272;0.092) | 3.37E-01 |  | -0.091 (-0.274;0.092) | 3.34E-01 |  | -0.151 (-0.366;0.064) | 1.68E-01 |
| Physical activity |  | males | -0.002 (-0.194;0.191) | 9.86E-01 |  | -0.005 (-0.198;0.187) | 9.57E-01 |  | -0.029 (-0.254;0.195) | 7.97E-01 |
| Physical activity | Severity of current depression | all | -0.343 (-0.502;-0.183) | 5.95E-05 |  | -0.341 (-0.501;-0.181) | 6.89E-05 |  | -0.409 (-0.601;-0.217) | 3.02E-05 |
| Physical activity |  | females | -0.422 (-0.631;-0.213) | 1.47E-04 |  | -0.419 (-0.628;-0.210) | 1.69E-04 |  | -0.534 (-0.800;-0.267) | 8.63E-05 |
| Physical activity |  | males | -0.254 (-0.451;-0.057) | 1.32E-02 |  | -0.255 (-0.453;-0.057) | 1.35E-02 |  | -0.191 (-0.469;0.087) | 1.79E-01 |
| Physical activity | Severity of anxiety | all | -0.106 (-0.254;0.041) | 1.61E-01 |  | -0.106 (-0.255;0.042) | 1.63E-01 |  | -0.153 (-0.333;0.027) | 9.60E-02 |
| Physical activity |  | females | -0.228 (-0.428;-0.029) | 2.72E-02 |  | -0.227 (-0.427;-0.026) | 2.92E-02 |  | -0.193 (-0.445;0.060) | 1.35E-01 |
| Physical activity |  | males | 0.039 (-0.143;0.221) | 6.74E-01 |  | 0.036 (-0.146;0.218) | 6.98E-01 |  | -0.058 (-0.307;0.190) | 6.45E-01 |

Table S10: Results of the inverse weighted (IVW) and pleiotropy robust Egger and Weighted median analysis using sedentary time as exposure.

|  |  |  | **IVW** |  |  | **Egger** |  |  | **Weighted Median** |  |
| --- | --- | --- | --- | --- | --- | --- | --- | --- | --- | --- |
| **exposure** | **outcome** | **strata** | **OR (95%CI)** | **P** |  | **OR (95%CI)** | **P** |  | **OR (95%CI)** | **P** |
| Sedentary | Current depression | all | 0.899 (0.598;1.351) | 6.11E-01 |  | 0.904 (0.601;1.358) | 6.29E-01 |  | 0.915 (0.514;1.630) | 7.63E-01 |
| Sedentary |  | females | 0.916 (0.549;1.529) | 7.39E-01 |  | 0.917 (0.549;1.531) | 7.42E-01 |  | 0.587 (0.281;1.225) | 1.56E-01 |
| Sedentary |  | males | 0.884 (0.404;1.936) | 7.60E-01 |  | 0.894 (0.410;1.949) | 7.80E-01 |  | 0.660 (0.250;1.746) | 4.03E-01 |
| Sedentary | Lifetime major depression | all | 1.064 (0.936;1.210) | 3.47E-01 |  | 1.062 (0.936;1.204) | 3.58E-01 |  | 0.984 (0.821;1.179) | 8.60E-01 |
| Sedentary |  | females | 1.089 (0.929;1.277) | 2.99E-01 |  | 1.089 (0.927;1.280) | 3.06E-01 |  | 0.982 (0.783;1.230) | 8.72E-01 |
| Sedentary |  | males | 1.015 (0.815;1.263) | 8.98E-01 |  | 1.007 (0.810;1.252) | 9.49E-01 |  | 1.008 (0.739;1.376) | 9.59E-01 |
| Sedentary | Current anxiety disorder | all | 0.906 (0.531;1.546) | 7.19E-01 |  | 0.916 (0.543;1.545) | 7.43E-01 |  | 0.925 (0.448;1.911) | 8.33E-01 |
| Sedentary |  | females | 0.669 (0.364;1.230) | 2.04E-01 |  | 0.672 (0.365;1.236) | 2.09E-01 |  | 0.664 (0.279;1.578) | 3.54E-01 |
| Sedentary |  | males | 1.556 (0.612;3.952) | 3.59E-01 |  | 1.589 (0.648;3.895) | 3.18E-01 |  | 1.241 (0.372;4.138) | 7.25E-01 |
| Sedentary | Lifetime anxiety disorder | all | 0.927 (0.720;1.193) | 5.58E-01 |  | 0.923 (0.717;1.189) | 5.38E-01 |  | 0.934 (0.656;1.330) | 7.03E-01 |
| Sedentary |  | females | 0.921 (0.670;1.266) | 6.15E-01 |  | 0.919 (0.668;1.263) | 6.04E-01 |  | 0.826 (0.526;1.299) | 4.09E-01 |
| Sedentary |  | males | 0.951 (0.609;1.485) | 8.27E-01 |  | 0.946 (0.606;1.475) | 8.07E-01 |  | 0.985 (0.517;1.876) | 9.64E-01 |
| **exposure** | **outcome** | **strata** |  |  |  |  |  |  |  |  |
| Sedentary | Well-being | all | -0.200 (-0.330;-0.070) | 4.60E-03 |  | -0.197 (-0.324;-0.070) | 4.20E-03 |  | -0.170 (-0.327;-0.012) | 3.44E-02 |
| Sedentary |  | females | -0.164 (-0.317;-0.010) | 4.30E-02 |  | -0.161 (-0.311;-0.011) | 4.27E-02 |  | -0.051 (-0.261;0.160) | 6.38E-01 |
| Sedentary |  | males | -0.246 (-0.431;-0.061) | 1.30E-02 |  | -0.244 (-0.429;-0.059) | 1.38E-02 |  | -0.189 (-0.430;0.052) | 1.25E-01 |
| Sedentary | Severity of major depression | all | 0.119 (-0.059;0.297) | 1.98E-01 |  | 0.116 (-0.059;0.290) | 2.02E-01 |  | -0.025 (-0.251;0.201) | 8.30E-01 |
| Sedentary |  | females | 0.080 (-0.135;0.295) | 4.71E-01 |  | 0.079 (-0.136;0.294) | 4.74E-01 |  | 0.050 (-0.256;0.356) | 7.47E-01 |
| Sedentary |  | males | 0.163 (-0.117;0.444) | 2.61E-01 |  | 0.157 (-0.115;0.430) | 2.64E-01 |  | 0.077 (-0.240;0.394) | 6.33E-01 |
| Sedentary | Severity of current depression | all | 0.138 (-0.078;0.354) | 2.18E-01 |  | 0.140 (-0.076;0.357) | 2.12E-01 |  | 0.230 (-0.062;0.522) | 1.23E-01 |
| Sedentary |  | females | 0.168 (-0.122;0.458) | 2.62E-01 |  | 0.172 (-0.117;0.462) | 2.51E-01 |  | 0.174 (-0.229;0.577) | 3.99E-01 |
| Sedentary |  | males | 0.099 (-0.203;0.402) | 5.23E-01 |  | 0.100 (-0.207;0.406) | 5.28E-01 |  | 0.195 (-0.218;0.608) | 3.55E-01 |
| Sedentary | Severity of anxiety | all | 0.019 (-0.163;0.201) | 8.38E-01 |  | 0.020 (-0.162;0.202) | 8.31E-01 |  | 0.097 (-0.159;0.354) | 4.58E-01 |
| Sedentary |  | females | 0.069 (-0.189;0.327) | 6.02E-01 |  | 0.069 (-0.189;0.327) | 6.01E-01 |  | 0.124 (-0.235;0.483) | 4.99E-01 |
| Sedentary |  | males | -0.005 (-0.275;0.265) | 9.70E-01 |  | -0.004 (-0.276;0.269) | 9.78E-01 |  | 0.094 (-0.280;0.469) | 6.21E-01 |

Table S11: Results of the MRLap analysis at different threshold for selecting exposure instrument.

|  |  |  | **5E-06 p-value threshold** |  |  |  | **1E-06 p-value threshold** |  |  |
| --- | --- | --- | --- | --- | --- | --- | --- | --- | --- |
| **exposure** | **outcome** | **strata** | **Beta (SE)** | **P** |  |  | **Beta (SE)** | **P** |  |
| Physical activity | Well-being | all | 0.096 (0.017;0.175) | 1.72E-02 |  |  | -0.012 (-0.115;0.092) | 8.22E-01 |  |
|  |  | females | 0.078 (-0.027;0.182) | 1.47E-01 |  |  | -0.03 (-0.168;0.109) | 6.76E-01 |  |
|  |  | males | 0.110 (0.014;0.207) | 2.54E-02 |  |  | 0.009 (-0.148;0.167) | 9.06E-01 |  |
|  |  |  |  |  |  |  |  |  |  |
| Physical activity | Severity of current depression | all | -0.096 (-0.174;-0.018) | 1.58E-02 |  |  | -0.026 (-0.138;0.086) | 6.51E-01 |  |
|  |  | females | -0.094 (-0.189;0.002) | 5.46E-02 |  |  | -0.031 (-0.169;0.108) | 6.64E-01 |  |
|  |  | males | -0.104 (-0.209;0.000) | 5.05E-02 |  |  | -0.031 (-0.187;0.124) | 6.94E-01 |  |
|  |  |  |  |  |  |  |  |  |  |
| Sedentary time | Well-being | all | -0.155 (-0.426;0.115) | 2.60E-01 |  |  | -0.192 (-0.543;0.16) | 2.85E-01 |  |
|  |  | females | -0.09 (-0.394;0.213) | 5.59E-01 |  |  | -0.121 (-0.525;0.283) | 5.58E-01 |  |
|  |  | males | -0.249 (-0.619;0.12) | 1.86E-01 |  |  | -0.312 (-0.698;0.074) | 1.13E-01 |  |

Table S12: Results of 2-sample MR analyses using physical activity and sedentary time as exposures and Wray et al., depression summary statistics as the outcome.

| **Exposure** | **MR Method** | **Odds of depression (95%CI)** | **P** |
| --- | --- | --- | --- |
| Physical Activity | IVW | 0.82 (0.74, 0.92) | 0.001 |
| Physical Activity | Egger | 0.77 (0.57, 1.04) | 0.10 |
| Physical Activity | WM | 0.82 (0.70, 0.97) | 0.018 |
| Physical Activity | PWM | 0.82 (0.70, 0.96) | 0.014 |
| Sedentary Time | IVW | 1.04 (0.88, 1.23) | 0.64 |
| Sedentary Time | Egger | 1.14 (0.71, 1.83) | 0.60 |
| Sedentary Time | WM | 0.98 (0.78, 1.24) | 0.89 |
| Sedentary Time | PWM | 0.98 (0.78, 1.24) | 0.89 |

IVW: Inverse variance weighted; WM: weighted median; PWM: penalised weighted median

Table S13: Results of the MRLap analysis of the individual items used in the mental health questionnaire (MHQ) in UK Biobank.

| **exposure** | **outcome** | **strata** | **OR (95%CI)** | **P** |
| --- | --- | --- | --- | --- |
| Physical activity | General happiness | all | 0.090 (0.027;0.152) | 4.90E-03 |
| Physical activity | General happiness | females | 0.098 (0.013;0.183) | 2.42E-02 |
| Physical activity | General happiness | males | 0.077 (-0.008;0.162) | 7.70E-02 |
| Physical activity | Happiness with health | all | -0.026 (-0.094;0.042) | 4.51E-01 |
| Physical activity | Happiness with health | females | -0.076 (-0.161;0.009) | 7.90E-02 |
| Physical activity | Happiness with health | males | 0.037 (-0.054;0.127) | 4.24E-01 |
| Physical activity | Meaningful life | all | -0.001 (-0.099;0.096) | 9.78E-01 |
| Physical activity | Meaningful life | females | 0.113 (-0.023;0.248) | 1.03E-01 |
| Physical activity | Meaningful life | males | -0.150 (-0.294;-0.006) | 4.14E-02 |
| **exposure** | **outcome** | **strata** | **OR (95%CI)** | **P** |
| Sedentary time | General happiness | all | -0.121 (-0.256;0.013) | 7.74E-02 |
| Sedentary time | General happiness | females | -0.068 (-0.249;0.113) | 4.60E-01 |
| Sedentary time | General happiness | males | -0.190 (-0.374;-0.006) | 4.26E-02 |
| Sedentary time | Happiness with health | all | -0.008 (-0.15;0.135) | 9.17E-01 |
| Sedentary time | Happiness with health | females | -0.054 (-0.258;0.150) | 6.04E-01 |
| Sedentary time | Happiness with health | males | 0.050 (-0.138;0.237) | 6.03E-01 |
| Sedentary time | Meaningful life | all | -0.074 (-0.276;0.128) | 4.72E-01 |
| Sedentary time | Meaningful life | females | -0.044 (-0.315;0.228) | 7.53E-01 |
| Sedentary time | Meaningful life | males | -0.095 (-0.416;0.225) | 5.60E-01 |
